# A Catalytic-Plasmonic Pt Nanoparticle Sensor for Hydrogen Detection in High-Humidity Environments

**DOI:** 10.1021/acssensors.5c03166

**Published:** 2025-11-18

**Authors:** Athanasios Theodoridis, Carl Andersson, Sara Nilsson, Joachim Fritzsche, Christoph Langhammer

**Affiliations:** Department of Physics, 11248 Chalmers University of Technology, SE 412 96 Gothenburg, Sweden

**Keywords:** hydrogen sensor, humidity, nanoplasmonic sensing, catalytic sensing, Pt nanoparticles

## Abstract

The detection of hydrogen gas in humid air environments is a key unresolved challenge for hydrogen safety in the rapidly growing hydrogen energy technologies, which hold a key to abate the CO_2_ emissions from particularly challenging sectors that together represent more than 20% of man-made CO_2_. In this work, we introduce a catalytic-plasmonic optical hydrogen sensor that employs nanofabricated and plasmonically active Pt nanoparticles as transducer elements for hydrogen detection in highly humid environments in air. Leveraging the combination of the Pt nanoparticles’ intrinsic high activity in the catalytic hydrogen oxidation reaction with their high sensitivity in plasmonic dielectric sensing, we show that this catalytic-plasmonic sensor is able to operate in the entire humidity range of 0–80% relative humidity accessible in our test setup and exhibits a measured limit of detection of 30–50 ppm hydrogen in air at 100 °C and 80 °C sensor operating temperatures, respectively, and that it delivers consistent and constant response to hydrogen during a 143 h long continuous measurement in 80% relative humidity. We also show that above a given hydrogen concentration, the sensor response magnitude to a specific hydrogen concentration increases with increasing humidity, which is the distinct opposite of any other known hydrogen sensing technology, whose response deteriorates or is entirely suppressed in high humidity. This advertises catalytic-plasmonic sensors as a new paradigm in the hydrogen sensor arena with particular promise for hydrogen detection in high-humidity environments.

## Graphical abstract

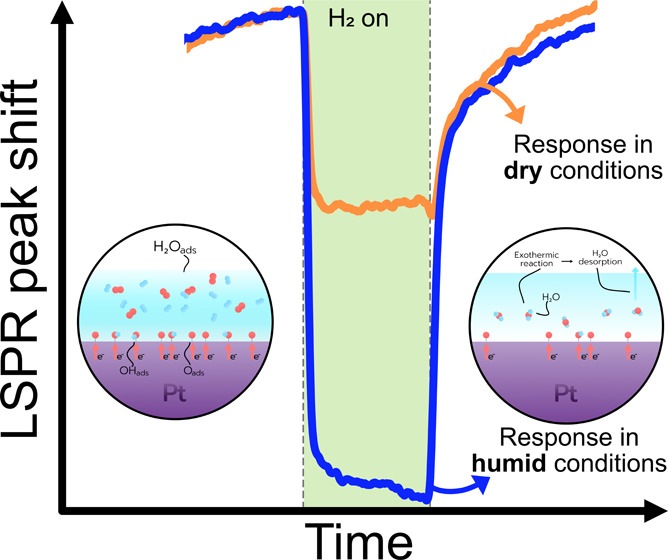

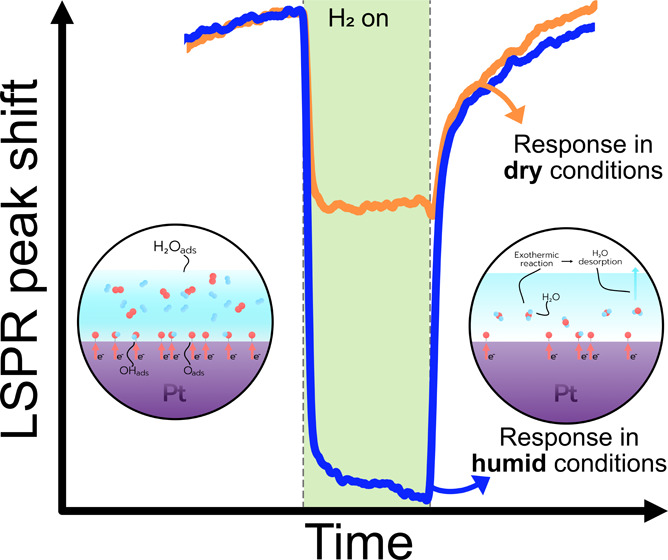

The hydrogen economy concept, which was coined in the 1970s,^1,2^ is today being realized at a rapidly increasing pace. This development is enabled by recent breakthroughs and advances in a multitude of enabling technologies, such as fuel cells and green hydrogen production, and the increasingly common insight that hydrogen (H_2_) holds one of the keys to significantly reduce CO_2_ emissions from particularly challenging sectors such as cement (replacement of fossil fuels used in heat kilns) and steel production (replacement of coke as reducing agent for iron ore), as well as heavy transport and shipping, that together represent more than 20% of man-made CO_2_.^3,4^ However, safety currently constitutes a barrier to the large-scale deployment of H_2_ technologies, especially when H_2_ is used in confined spaces with limited venting possibilities and/or close to public areas and people’s homes, where the risk for H_2_-related accidents due to the flammability of H_2_-air mixtures increases substantially with potentially catastrophic consequences. Hence, to safely implement H_2_ technologies without delays, mitigating these risks is imperative and underpins the importance of H_2_ safety sensors. Equally important are sensors for process monitoring in *e.g*., H_2_ combustion, electricity production, fuel cells, and electrolyzers to optimize these processes.

For this reason, research is directed toward the development of H_2_ sensor technologies with operational principles ranging from electrochemical^5,6^ to catalytic,^7–9^ thermal conductivity,^10–12^ and optical,^13,14^ all aimed at meeting performance targets set by different stakeholders, with the most stringent ones focusing on safety defined by the US Department of Energy (DoE).^15–17^ Such targets typically include the response/recovery time of a sensor, its limit of detection, accuracy, and lifetime. In addition to these intuitive sensor metrics, the reliable operation of H_2_ sensors in demanding chemical environments is equally important but significantly less investigated to date. One such environment of particular technical importance that is little understood are high or fluctuating humidity levels. Its practical importance stems from the fact that, for example, the gas feed in certain fuel cell systems is humidified to enable stable operation of the polymer exchange membrane or that humidity can fluctuate widely in open environments due to weather and geographical factors. Accordingly, H_2_ sensor performance targets for humid environments encompass 20 to 80% relative humidity (RH) for stationary hydrogen applications^18,19^ and 0 to 100% RH for automotive systems.^17,19^

Despite the obvious importance, research in this direction is very limited and the few studies that address the “humidity challenge” either only do so partially, *i.e*., for a narrow humidity range or generally low RH levels, or at conditions that are limiting from the application perspective.^8,20–24^ An example for the former category is a resistivity sensor that employs an indium–tin oxide (ITO) sensing layer decorated with colloidal Pd–Ni or Pt nanoparticles stabilized by a highly hydrophilic surfactant and that exhibits excellent sensing metrics but only up to 60% RH.^23^ An example for the latter category are SnO_2_ and In_2_O_3_ thin film sensors that have been demonstrated to exhibit a limit of detection (LoD) down to 25 ppm of H_2_ and compatibility with a wide humidity range of 20–95% RH, but at a cost of a sensor operating temperature of 623 K.^24^ As a second interesting example is the work by Geng et al.,^8^ who employed grain-boundary rich colloidal Pt nanoparticles drop-casted onto a thermocouple for thermocatalytic H_2_ sensing based on the exothermic H_2_ oxidation reaction (HOR)^25^ in a wide humidity range of 0–98% in air, but with a significantly deteriorating LoD for increasing RH, reaching an unsatisfying LoD of 3% H_2_ at 98% RH.

From a general mechanistic perspective, the key challenge with detecting H_2_ in humid air environments is the fact that, at ambient conditions and temperatures, the sensor surface is covered by (multilayers of) H_2_O and OH species,^26,27^ as well as O, possibly together with trace amounts of other molecules abundant in air, such as CO, NO_*x*_, or SO_*x*_. Since almost all H_2_ sensors critically rely on a (dissociative) chemical interaction of H_2_ molecules with the sensor surface, these H_2_O and OH species effectively block sizable fractions of the sites on the surface necessary for H_2_ to chemisorb and dissociate and thereby trigger a sensor signal (the exact amount of “blocking” depends on the specific system and conditions at hand). Typical consequences of this blocking effect range from increasing response times to reduced LoDs, to shifting baselines, to complete sensor deactivation.

In this work, to overcome the current limitations of H_2_ sensing in humid air environments of particular importance for safety applications, we introduce a combination of the highly sensitive optical nanoplasmonic (H_2_) sensing principle^15,28–30^ with catalytic H_2_ detection on Pt nanoparticles. Specifically, we demonstrate a catalytic-plasmonic Pt nanoparticle H_2_ sensor that leverages the dielectric plasmonic sensing capabilities of Pt nanoparticles^29^ in concert with Pt surfaces’ ability to catalyze the hydrogen oxidation (HOR) reaction and the corresponding changes in inelastic electron scattering depending on the surface coverage of O and H species.^31,32^ This sensor can operate in the entire humidity range of 0–80% RH accessible in our test setup and exhibits a measured LoD significantly below the DoE target (<1000 ppm) for all operating temperatures investigated. Specifically, the sensor exhibits a measured LoD of 30–50 ppm in air at 80 °C operating temperature and above and throughout the entire humidity range. Furthermore, as a second remarkable key result, we reveal that the sensor response magnitude above a specific hydrogen concentration (depending on the temperature of operation as we discuss in detail below) *increases* with increasing humidity, rendering it most sensitive to H_2_ at the highest humidity levels. This is a stark contrast to all other known H_2_ sensor technologies, whose H_2_ sensitivity dramatically deteriorates when humidity increases. Finally, our long-term sensor stability investigation over more than 143 h at 80% RH and 80 °C operating temperature in synthetic air highlights the robustness of the sensor over an extended period of time, and selectivity/deactivation tests at 80% RH in synthetic air involving the flammable gases CH_4_ and CO according to the ISO 26142:2010 ^18^ standard, as well as C_3_H_6_, reveal high selectivity and deactivation resistance in particular at low H_2_ concentrations.

## Results and Discussion

### Designing a Pt Nanoparticle Catalytic-Plasmonic Hydrogen Sensor

In this work, we combine the concepts of nanoplasmonic and catalytic sensing in a single device (see SI Sections 1 and 2 for a more detailed introduction of these two sensing principles), using a nanostructured Pt surface. Specifically, using Hole-Mask Colloidal Lithography (HCL),^33^ we nanofabricated a quasi-random array of Pt nanodisks onto a fused silica substrate (Figure 1a, see the Methods section for details). The nanodisks have an average diameter of 215 ± 10 nm at a constant nominal height of 25 nm, and they are characterized by a high degree of polycrystallinity after the physical vapor deposition of the Pt through the nanolithography mask (Figure 1a, transmission electron microscopy (TEM) image inset, Supporting Figure S2 for SEM images and size analysis). To assess the LSPR of this surface, we exhibited an optical extinction measurement using a spectrophotometer (Figure 1b), revealing a broad but distinct LSPR peak with a maximum at ∼710 nm (Figure 1c), in good agreement with the literature.^34^ To subsequently evaluate the H_2_ sensing properties of this Pt nanoparticle surface, we employed a heatable quartz tube catalytic plug-flow reactor with optical access for plasmonic measurements in optical extinction configuration using fiber optics, a halogen lamp, and a fixed grating spectrometer (Figure 1d, see the Methods section for details). This reactor is connected to mass flow controllers (MFCs) for the accurate steering of the gas environment as well as to a controlled evaporator mixer (CEM) for humidity control and a humidity probe for accurate humidity measurements. We highlight that the supplied water flow through the liquid flow controller (LFC) corresponds to RH values referenced to the 30 °C humidifying temperature of the gas. Therefore, when the humidified gas is admitted to the reactor chamber, where temperatures may be higher, the relative humidity will be lower. We chose this scenario to emulate the operation of a H_2_ safety sensor, whose active surface is typically heated, at ambient conditions.

**Figure 1:**
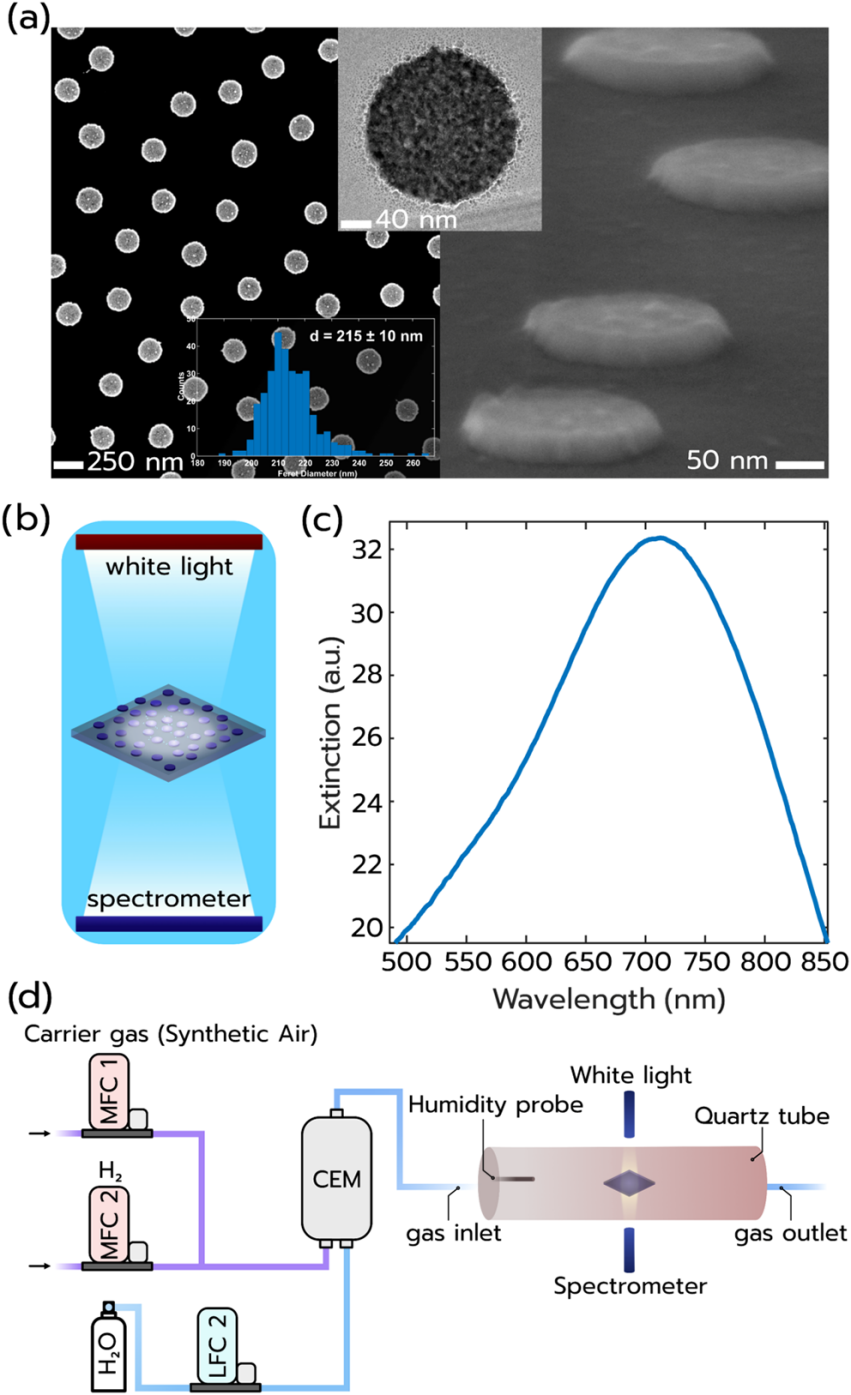
(a) Top- and side-view SEM images of the quasi-random Pt nanodisk array used as a catalytic-plasmonic H_2_ sensor. The first inset depicts a TEM image of a single Pt nanodisk and reveals its highly polycrystalline morphology. The second inset shows the nanodisk diameter histogram obtained from SEM image analysis and reveals an average diameter of 215 ± 10 nm. (b) Schematic depiction of an optical extinction measurement of the Pt nanodisk quasi-random array. (c) Optical extinction spectrum of the Pt nanodisk quasi-random array, revealing a broad but distinct LSPR peak with a maximum at ∼710 nm. (d) Schematic depiction of the experimental setup used to assess the H_2_ sensing properties of the Pt nanodisk quasi-random array sensor surface. The synthetic air carrier gas is first blended with the desired H_2_ concentration using mass flow controllers (MFCs). This mix is subsequently humidified in a controlled evaporator mixer (CEM) before it enters the quartz tube plug-flow reactor with optical access used to continuously track the LSPR peak of the Pt nanodisk sensor sample mounted inside the reactor.

As the next step, to first investigate the response of the Pt nanoparticle sensor surface to varying RH in synthetic air and in the *absence* of H_2_, we executed a humidity titration experiment from 0% RH to 80% RH (the highest humidity level attainable in our setup) at 33 °C, 50 °C, 80 and 100 °C sensor operating temperature, respectively (Figure 2a–c). Evidently, there is a distinct red-shift in the spectral position of the LSPR peak maximum, λ_peak_, upon increasing the humidity from 0% RH to 10% RH for all sensor operating temperatures, with the change being largest at the lowest temperature of 33 °C (Figure 2a). Further increasing the RH results in additional red-shifting of λ_peak_ at all temperatures, however, with magnitudes distinctly smaller than those for the first RH step but at the same time proportional to the RH (Figure 2c). This results in spectral shifts, Δλ_peak_,^35^ of up to 3.7 nm at 80% RH and 33 °C sensor operating temperature, which are easily resolved even by the naked eye (Figure 2b). Notably, this response is reversible upon reduction of the RH, for all sensor temperatures (Figure 2a,c). We attribute the observed spectral shifts of λ_peak_ to the RH-dependent condensation of water mono- and multilayers on the sensor surface,^36–38^ whose thickness, for a given RH, decreases as sensor temperature increases since we, as described above, use RH values referenced to the 30 °C humidifying temperature of the gas. These water layers increase the RI in the close vicinity of the Pt particles, which is expected to spectrally red-shift their LSPR in proportion to their thickness,^34,39^ as confirmed by corresponding Finite-Difference Time-Domain (FDTD) simulations (Supporting Figure S3).

**Figure 2:**
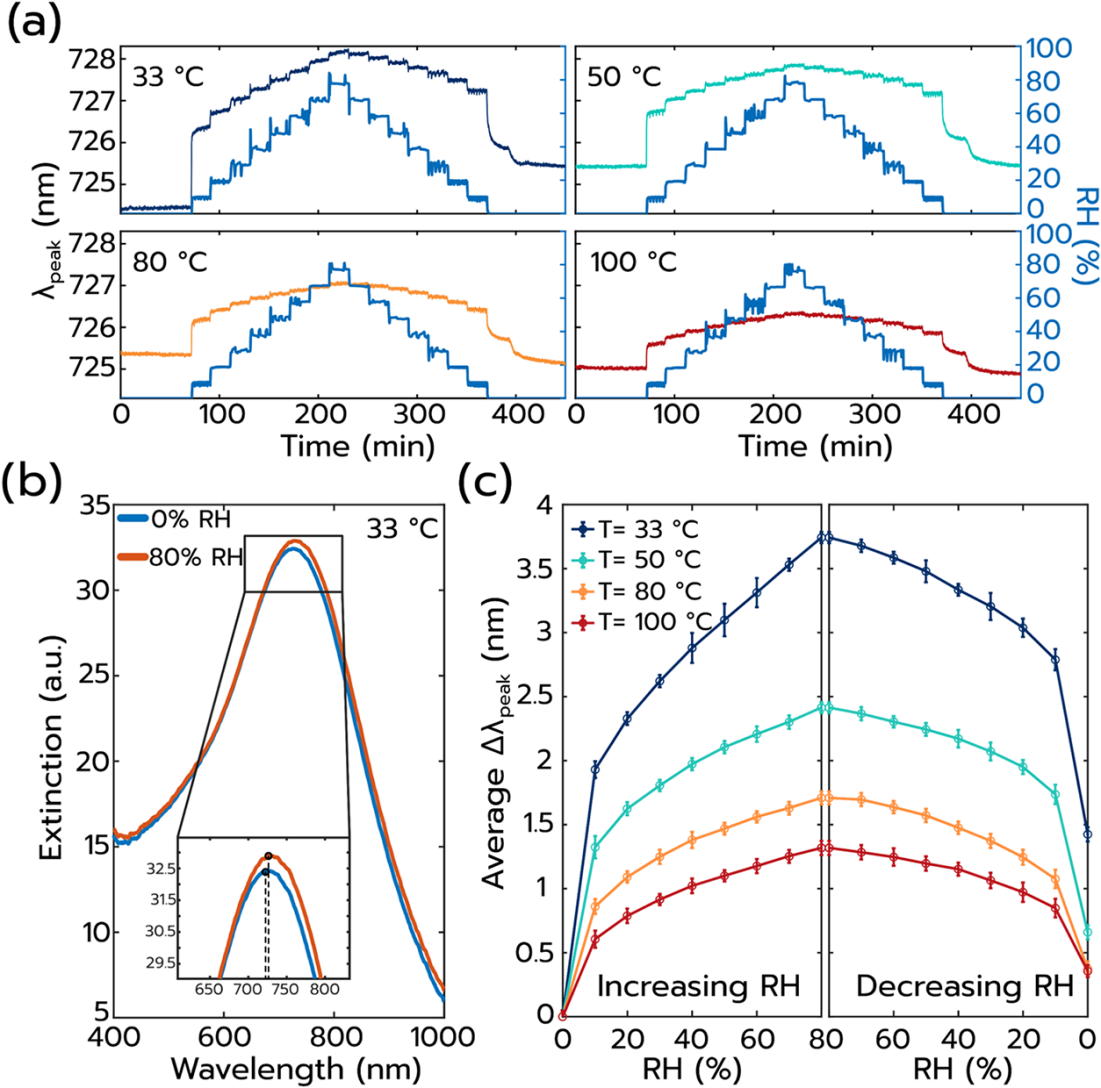
Humidity titration experiment on the Pt sensor surface in synthetic air in the absence of H_2_. (a) Δλ_peak_ measured for RH in/decrease in 10% RH increments at 33, 50, 80, and 100 °C sensor operation temperature, plotted together with the humidification levels of the gas stream measured at 30°, prior to admission of the humidified gas to the measurement chamber. Prior to each measurement at a given sensor operation temperature, the sensor was exposed to dry synthetic air for 155 min. (b) Representative optical extinction spectra of the Pt sensor obtained at 0% and 80% RH, at a sensor operation temperature of 33 °C. (c) Extracted average Δλ_peak_ (mean value of 60 data points, at each RH step) versus RH for all sensor operating temperatures. Error bars correspond to 3 times the standard deviation (3σ).

### A Catalytic-Plasmonic H_2_ Sensor

Having established the Pt sensors’ response to different levels of RH in the absence of H_2_, it is now interesting to map the Δλ_peak_ response to different H_2_ concentrations, at different levels of RH, and at different sensor operation temperatures (for a quantitative analysis of the activity of the Pt sensor surface in the HOR, see SI Section 5). For this purpose, we established a measurement protocol comprised of symmetric H_2_ concentration pulse sets starting at 0.06 vol % H_2_ (600 ppm, ppm) and increasing to 0.13, 0.28, 0.60, and finally to 1.26% H_2_, and decreasing back down to 0 vol % H_2_ (Figure 3a). These sets are repeated 5 times at each sensor operation temperature, *i.e*., first in dry synthetic air at 0% RH, followed by 20, 50, and 80% RH, and finalized in dry synthetic air after an 11.5 h long dwell to desorb excess water from the sensor surface. Extracting the Δλ_peak_ response of the sensor to this protocol for the 5 different RH scenarios reveals that Δλ_peak_ blue-shifts (i.e., shifts to shorter wavelengths) for all H_2_ pulses at all conditions (Figure 3b and Supporting Figure S5). We highlight that this observed Δλ_peak_ to shorter wavelengths upon H_2_ exposure is the direct opposite to what is observed for hydride-forming Pd-based H_2_ sensors which exclusively exhibit Δλ_peak_ to longer wavelengths, *i.e*., spectral red-shifts.^15^ This clearly indicates a different sensing mechanism at play here, as expected for Pt that neither forms interstitial hydrides nor solid solutions with hydrogen under ambient conditions. We also note that the Δλ_peak_ blue-shifts observed are opposite to Δλ_peak_ in the H_2_O titration experiments above (***cf***. Figure 2).

**Figure 3:**
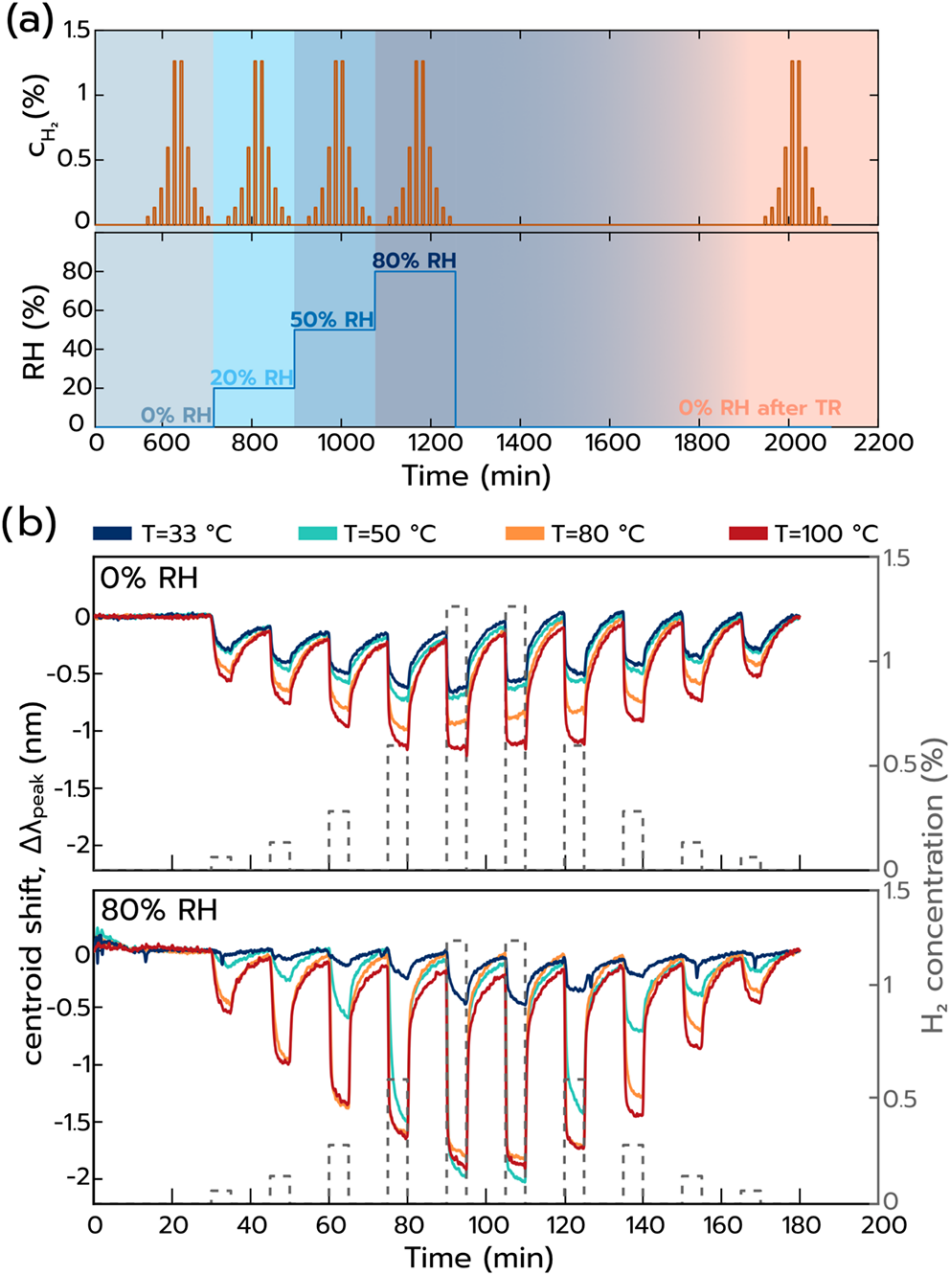
(a) General gas protocol used for the H_2_ sensing performance measurements. Four sets of H_2_ pulses, ranging from 0.06 to 1.26 vol % are introduced in the reactor at varying relative humidity values (0–80% RH). In all of the measurements, the background gas is synthetic air, and the temperature in each measurement is kept constant along this entire protocol (4 different measurements at *T* = 33, 50, 80, 100 °C). “TR” stands for temperature regeneration, where after the highest RH value, the sensor was exposed for 11.5 h to a constant background gas flow, at 0% RH for each corresponding temperature. (b) Sensor performance over time at 0% and 80% RH, for 4 different temperatures (***cf***. Supporting Figure S5 for 20, 50, and 0% RH after TR). The left *y*-axis shows the shift of the centroid, normalized to the value at the beginning of each set of pulses. The right *y*-axis shows the hydrogen concentrations ranging from 0.06 to 1.26 vol %.

To analyze the Pt sensor response in more detail, we start at 0% RH. Under these conditions, we notice a H_2_ concentration-dependent reversible response, whose magnitude for a given H_2_ concentration also depends on temperature, where higher temperatures yield a larger Δλ_peak_ (Figure 3b). Increasing RH to 20 and 50% yields similar responses and trends, but with larger Δλ_peak_ amplitudes as the main difference (Supporting Figure S5). Further increasing RH to 80% (Figure 3b) confirms the above and magnifies the Δλ_peak_ amplitudes for a given H_2_ concentration pulse. Remarkably, this result means that in stark contrast to all other reported H_2_ sensors, here the signal amplitude increases for increasing RH.

Based on the above results, it is now interesting to derive a sensing mechanism (Figure 4). To do so, we first consider the situation under ideal dry conditions in air. O_2_ will dissociatively chemisorb on Pt and, due to the high electronegativity of the chemisorbed O species,^25,40^ reduce the free electron density in the Pt nanoparticle (and thus decrease its Fermi level and increase its work function ϕ), which induces a spectral red-shift of the LSPR compared to a Pt particle with an O-free clean and dry surface (Figure 4a).^41–43^ Admitting H_2_ to the system in this state, *i.e*., a Pt nanoparticle with O-covered surface, will replace a H_2_ partial pressure-dependent fraction of the chemisorbed O by dissociatively chemisorbed and electropositive H species.^44^ Hence, the free electron density of the Pt nanoparticle increases (the Fermi level increases and work function ϕ decreases) and the LSPR is blue-shifted.^41–43^ Increasing the H_2_ partial pressure is thus expected to further increase this blue-shift, as experimentally, in fact, observed in our earlier work.^45^ A second aspect to consider as soon as H_2_ is admitted to this system is the HOR, which will generate chemisorbed hydroxyl (OH) species on the Pt surface as intermediate species in the H_2_O formation and thermal desorption process. From an electronegativity perspective, OH on Pt is slightly less electronegative than O.^46^ Hence, the catalytic transformation of these species to desorbing H_2_O, in principle, is also accompanied by a spectral blue-shift of the LSPR. However, since OH formation and reaction are in equilibrium if the system is in a steady state, the dominating contribution to an electron density change of the Pt particle is H-chemisorption-induced reduction of the O coverage.

**Figure 4:**
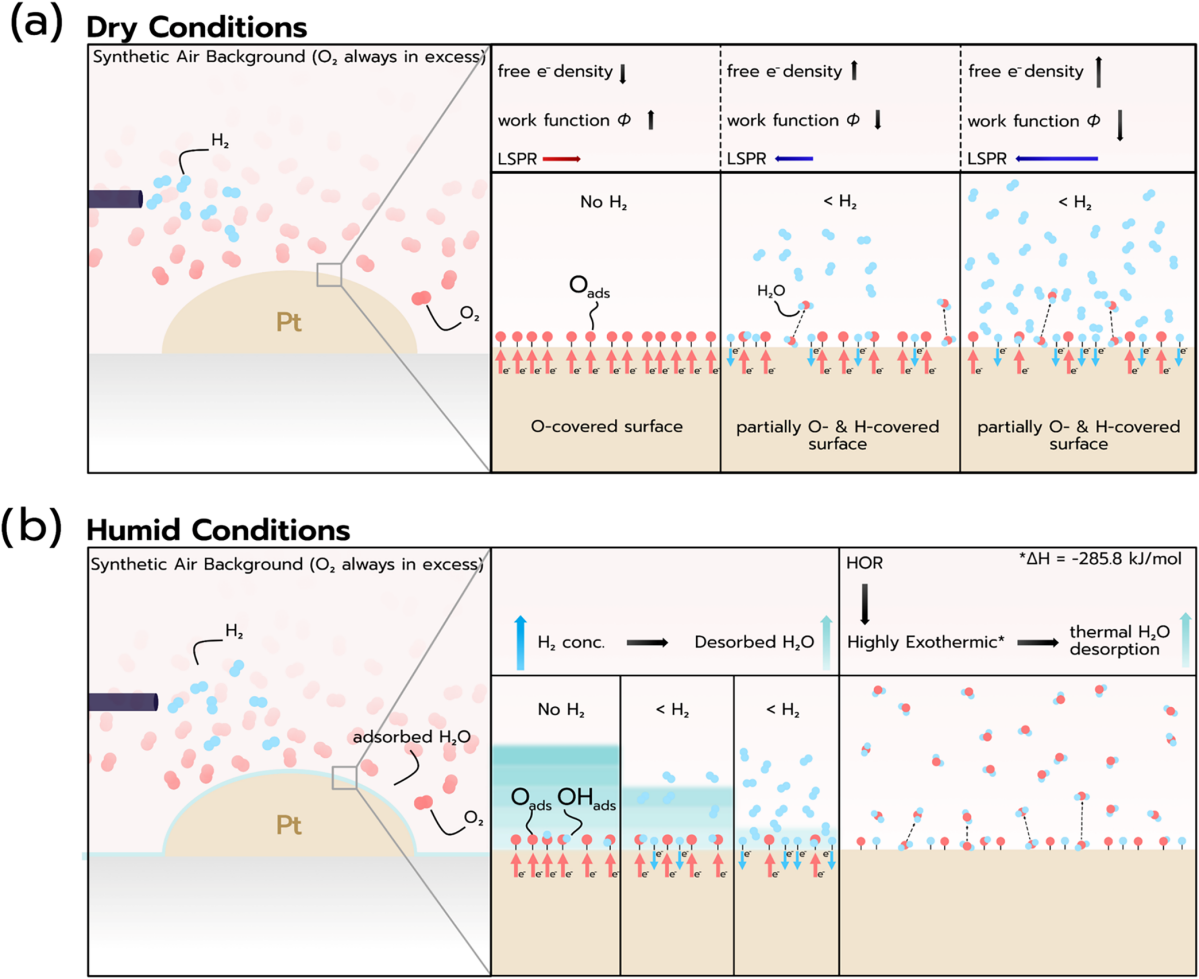
Schematic illustration of the sensing mechanism. (a) In dry conditions and in the absence of H_2_, the surface will be covered by dissociated O atoms due to the constant flow of synthetic air. Due to its high electronegativity, O will attract electrons from the surface, decreasing the free e^–^ density, increasing the work function (Φ), and red-shifting the LSPR. In the presence of H, the O-covered surface will be partially replaced by dissociated H atoms and OH groups. The H and OH species are less electronegative than O (H is, in fact, electropositive with respect to Pt) and therefore lead to an increase in the free e^–^ density, a decrease in Φ, and a blue-shift of the LSPR. Increasing the H_2_ concentration leads to further blue-shift of the LSPR. (b) Under humid conditions, the surface will be additionally covered by multilayers of H_2_O both molecularly and dissociatively adsorbed, depending on the specific site. In the absence of H_2_, the O and OH species adsorbed induce a red-shift of the LSPR (as described for the dry conditions) and the presence of H_2_O further leads to a thickness-dependent red-shift due to the dielectric plasmonic sensing mechanism. Now in the presence of H_2_, a blue-shift of the LSPR occurs, deriving both from the partial replacement of O and OH species on the Pt surface (as described for the dry conditions), and the highly exothermic HOR, which leads to thermal desorption of adsorbed H_2_O.

Turning to humid conditions in air (and thus O_2_) first *without* H_2_ (Figure 4b), we remember that (i) increasing RH leads to the formation of H_2_O layer(s) on the Pt nanoparticle surface whose thickness is RH- and temperature-dependent (***cf***. Figure 2), as well as (ii) to the dissociation of H_2_O into −OH and −H species, chemisorbed on the sensor surface, whose extent depends on the predominant surface facets, which in our case are expected to be low index with a high abundance of edge and step sites at grain boundaries (***cf***. Figure 1),^27,47,48^ (iii) the presence of chemisorbed O (from air), and OH and H (from dissociated H_2_O) reduces the electron density of the Pt and thus induces a spectral red-shift of Δλ_peak_,^41,42^ (iv) the water layer formation also leads to a thickness-dependent red-shift of Δλ_peak_ via the dielectric plasmonic sensing mechanism (***cf***. Figure 2). In other words, and as a key point here, mechanisms (iii) and (iv) are additive in terms of Δλ_peak_ red-shift. Adding also H_2_ to the system, molecular H_2_ diffuses through the water layers,^49^ dissociates and reduces the O and OH surface coverage, and induces the highly exothermic HOR (Δ*H* = −241.8 kJ/mol),^50^ whose rate depends both on temperature and H_2_ concentration. In concert, in the corresponding experiment (***cf***. Figure 3), this induces a distinct blue-shift of Δλ_peak_ when hydrogen pulses are applied, whose magnitude increases for increasing RH and a given H_2_ concentration in the pulse.

This leads to the following proposed sensing mechanism of our catalytic-plasmonic Pt H_2_ sensor in humid conditions (Figure 4b). In the absence of H_2_, and thus in the absence of the HOR, the Pt sensor surface is covered with water layers with a thickness proportional to the RH and temperature. At the atomic level, the Pt surface is covered with dissociated O from air, and with OH and H from dissociated H_2_O, as well as with molecularly adsorbed H_2_O, depending on the specific site. When this system is subsequently exposed to a H_2_ pulse, molecular H_2_ diffuses through the water layer, dissociates on the Pt surface, and reacts with dissociated O supplied by the synthetic air background according to the HOR mechanism (or with OH from dissociated H_2_O). Since the HOR is very fast, as well as highly exothermic on Pt, the dissipated reaction heat sizably increases the temperature of the Pt particles,^51,52^ which induces the (partial) thermal desorption of both H_2_O formed as a product of the HOR, and of the already present H_2_O in the water layer on the sensor surface. This hypothesis is also supported by identical location transmission electron microscopy (IL-TEM) showcasing the temporal evolution of the Pt particles’ morphology that is characterized by a significant degree of surface recrystallization upon continuous exposure to varying H_2_ pulses and humidity levels, as a result of the high exothermicity of the HOR (Supporting Figure S1). Additionally, the thermal desorption of H_2_O is expected to be more pronounced with increasing surface coverage of Pt particles on the sensor’s surface, due to the larger amount of active sites per area and possibly even a collective heating effect.^53–56^ To this end, our investigation showed that more densely populated sensor surfaces indeed yield more efficient thermal desorption of H_2_O and therefore a stronger signal response to a given H_2_ pulse (for more details, see SI Section 9).

Since the rate of the HOR, and thus the amount of dissipated reaction heat, is proportional to the H_2_ concentration in the pulse (Supporting Figure S4), higher H_2_ concentrations lead to more efficient H_2_O desorption from the water layer. This explains both the spectral blue-shift of Δλ_peak_, its H_2_ concentration dependence at a given RH, and its largest magnitude observed for the highest RH at which the initially adsorbed water layer is the thickest. Mechanistically, the blue-shift is the combination of (i) the dielectric plasmonic sensing mechanism, where the reduced water layer thickness changes the local RI around the plasmonic Pt particles, and (ii) the electron density change mechanism mediated by the change in electronegative O coverage on the Pt surface.

To further corroborate this sensing mechanism based on the overview analysis of the sensor performance presented in Figure 3b, we have extracted Δλ_peak_ as a function of H_2_ concentration for the different sensor operating temperatures and RH values (Figure 5a–d and Supporting Figure S6). Focusing first on the lowest temperature of 33 °C and 0% RH, we find Δλ_peak_ ∼ 0.3 nm for the lowest H_2_ concentration pulse (0.06%) followed by a very weak H_2_ concentration dependence of Δλ_peak_ (Figure 5a). According to the proposed sensing mechanism, this is likely the combination of the electron density change induced by H_2_ introduction and to a lesser extent the desorption of a very thin water layer likely present due to (i) the trace amounts of H_2_O in the synthetic air background (1 ppm according to specification) and (ii) the fact that the reactor is not completely sealed, which leads to diffusion of small amounts of ambient (humid) air into the reactor system and thus adsorb on the sensor surface. At 20% RH, an almost linear dependence of Δλ_peak_ on H_2_ concentration, with a maximal Δλ_peak_ of 1.7 nm for 1.26% H_2_ and a Δλ_peak_ ∼ 0.25 nm for the lowest H_2_ concentration, is apparent, which is very similar to the response in dry conditions. Interestingly, increasing RH to 50% yields a similarly linear but generally slightly smaller Δλ_peak_ response at all hydrogen concentrations. Further increasing RH to 80% further generally reduces the Δλ_peak_ response across the board and below the levels recorded in dry synthetic air (0% RH). Yet, a close to linear dependence of Δλ_peak_ on the H_2_ concentration is still observed. This deteriorated response for higher RH at 33 °C likely finds it origin in the corresponding larger thickness of the water layer on the surface at these conditions, hampering the diffusive supply of H_2_ from the gas phase through that water layer, and thus reducing the net H coverage on the surface (and thus the electron density change contribution to Δλ_peak_) and rendering the HOR mass transport limited and (in part also due to the low HOR catalytic rate at this low temperature) unable to completely thermally desorb the water layer. This, in turn, minimizes the dielectric part of the sensor response.

**Figure 5:**
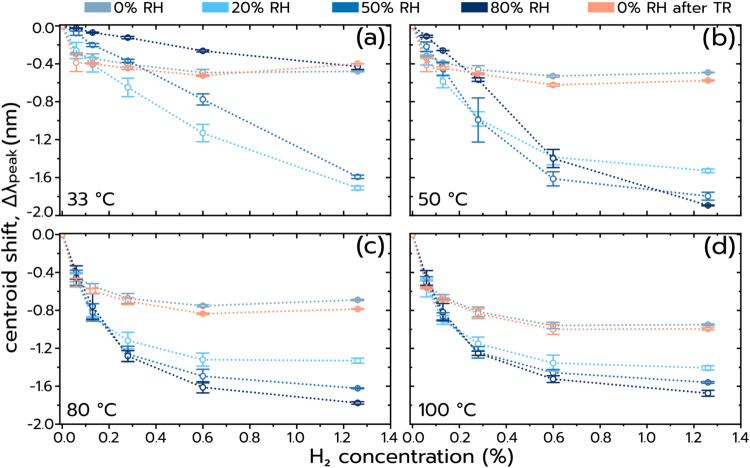
Centroid shift, Δλ_peak_, as a function of H_2_ concentration at (a) 33 °C, (b) 50 °C, (c) 80 °C, and (d) 100 °C. Each panel depicts 5 curves corresponding to 0% RH (at the beginning and after TR), 20% RH, 50% RH, and 80% RH. The H_2_ concentration was varied in steps from 0.06 to 1.26 vol % H_2_ in synthetic air background. Each point corresponds to the average between 2 identical H_2_ pulses within the same pulse set, where the error bar corresponds to the difference between them.

Increasing the sensor operation temperature to 50 °C reveals an almost identical Δλ_peak_ at 0% RH as observed at 33 °C (the slight increase finds its likely cause in a slightly shifted equilibrium between O and H on the surface), which corroborates that this signal predominantly stems from the electron density change (Figure 5b). Increasing the RH to 20% at 50 °C, however, reveals distinct differences as the Δλ_peak_ response is no longer linear in H_2_ concentration and increases more rapidly already at lower H_2_ concentrations. The sensor then responds very similarly also at 50 and 80% RH, however, still with a generally slightly smaller Δλ_peak_ magnitude at 80% RH. These observations are in line with a higher HOR rate at 50 °C compared to 33 °C and thus an accelerated desorption of parts of the H_2_O layer already at lower H_2_ concentrations, in line with the dielectric sensing mechanism now being the dominating one.

Moving to 80 °C, we find that the response magnitude at 0% RH has increased to Δλ_peak_ ∼ 0.45 nm for the lowest H_2_ concentration pulse, which we can assign to a temperature-induced shift in the equilibrium O and H coverage on the Pt surface in favor of H and the corresponding larger change in electron density (Figure 5c). For increasing RH, we observe a consistently larger response the higher the RH for H_2_ concentrations above 0.13%. We also notice that the Δλ_peak_ value appears to converge toward a saturation level for H_2_ concentrations above the 1.26% investigated here. These observations are in line with a predominate dielectric sensing mechanism that indeed implies that (i) once the complete H_2_O layer is desorbed by the HOR heat (which is expected at the highest H_2_ concentration since H_2_ is in deficit by 1 order of magnitude compared to O_2_), no further sensor response is to be expected and (ii) that maximum Δλ_peak_ value is the largest for the highest RH as the desorbed water layer was the thickest.

Finally moving to 100 °C, we observe a largely very similar response to that at 80 °C, as expected according to the two sensing mechanisms at play (Figure 5d). However, we also notice one additional fact that the absolute Δλ_peak_ values at 100 °C for H_2_ concentrations larger than 0.13% are somewhat smaller compared to 80 °C and even smaller compared to 50 °C. This can be understood as the interplay between a higher HOR rate at higher temperatures, which boosts the sensor’s ability to desorb the water layer, and a generally reduced thickness of the water layer at higher temperatures, which will lower the maximally possible Δλ_peak_ response (***cf***. Figure 2). Hence, this interplay implies that an optimal sensor operation temperature exists as the best compromise between these two effects. From our data, it becomes clear that 80 °C constitutes this optimal sensor operation temperature for H_2_ detection in high-humidity environments in air as it delivers the largest Δλ_peak_ response across the board of studied H_2_ concentrations, with a so far measured LoD of 0.06% or 600 ppm of H_2_ at 80% RH in air. However, our data also imply that a significantly lower LoD is likely possible as the measured Δλ_peak_ response at 0.06% H_2_ is significantly above the noise level (***cf***. Figure 3b). To verify that and measure the true LoD for each temperature of operation, we have exposed the same sensor to H_2_ concentrations down to 0.003 vol % or 30 ppm, at the same temperatures and RH levels (Supporting Figure S7). Remarkably, the sensor exhibits an LoD far below the DoE target of 1000 ppm for the entire investigated humidity range (0–80%), and even at the lowest temperature of 33 °C. Focusing on the above-identified optimal operation temperature of 80 °C, the sensor exhibits a measured LoD of 30 ppm in 0–50% RH, and 50 ppm in 80% RH, which is more than 1 order of magnitude below the DoE target even at the highest RH investigated (for more details, see SI Section 8). In fact, we argue that, to the best of our knowledge, the reported sensor exhibits the lowest operating temperature and LoD at 80% RH in air, without the use of advanced machine learning-based data treatment^57,58^ (Supporting Table S1). As a final remark, we have also analyzed the sensor’s response/recovery times and found that they meet the ISO 26142:2010 ^18^ response time target (*t*_90_ < 30 s) for 0.6 vol % H_2_ at 20% RH, and the recovery target (*t*_10_ < 60 s) for all measured RH and H_2_ concentrations. Notably, and as elaborated in detail in SI section 10, these metrics were achieved under far from ideal conditions in a reactor with a gas exchange time constant of close to 1 min, meaning that the intrinsic response/recovery times of the sensor likely are significantly faster.

### Selectivity/Poisoning Resistance and Long-Term Sensor Stability

As a final step, we investigated the robustness of the catalytic-plasmonic sensor, when subjected to challenging conditions, by performing both selectivity/poisoning measurements in the presence of interfering gases and a long-term stability measurement over 143 h in total. For the selectivity/poisoning measurements, the sensor was exposed to 3 different interfering gases, namely, the flammable gases CH_4_ and C_3_H_6_, and CO. The sensor exhibits substantial poisoning resistance and high selectivity to H_2_, especially in the low H_2_ concentration regime, useful in early leak detection in dry but also highly humid environments (for more details, see SI section 11). For the long-term stability measurement, the sensor was exposed to multiple regular and randomized H_2_ pulse sets including 0.06, 0.13, 0.28, 0.60, and 1.26 vol % H_2_ concentration pulses (see the Methods section for details) at 80% RH for 114 h, where the selected operating temperature was the above-identified optimal 80 °C (Figure 6a). The correspondingly obtained λ_peak_ response of the sensor as a function of time reveals the following key observations (Figure 6b): (i) at the end of the dry stage, denoted by the second dashed line, λ_peak_ suddenly red-shifts, due to the introduction of H_2_O, as expected according to the dielectric sensing mechanism and with the humidity titration experiment (***cf***. Figure 2). (ii) A distinct and repeatable response of the sensor to the applied H_2_ pulses in both the random and regular pulse sets. (iii) A repeatable baseline drift, starting from ∼20h into the measurement (first H_2_ pulse set) and until the ∼38h mark, which repeats 3 times, until the 86h mark, beyond which the baseline becomes stable. This can be explained by daytime/nighttime/weekend-related changes in the ventilation conditions in our lab (see Supporting Figure S18 for details). To, as the final analysis step, visualize the quantitative reproducibility of the sensor’s response to specific H_2_ concentrations within this experiment, we extract Δλ_peak_ values for the 0.06, 0.28, and 1.26 vol % H_2_ pulses within all regular pulse sets, and plot them together (Figure 6c). Since in every pulse set each H_2_ concentration is present twice, we choose to always plot the second pulse to avoid any influence of the time that the sensor had spent in synthetic air, prior to H_2_ exposure in a specific pulse set (see the Methods section for more details). For all three concentrations, we note a consistent and highly repeatable response to H_2_. Executing the same type of analysis for the randomized pulse sets essentially yields the same result as a highly repeatable sensor response (Figure 6d). In summary, this showcases the robustness and stability of the Pt nanoparticle catalytic-plasmonic H_2_ sensor platform we have introduced in this work, even when operating under the most challenging humidity conditions of 80% RH that we can generate in our setup for a total of 143 h. As a final remark, we also highlight that, in fact, this sensor had already been exposed to a large number of similar experiments at varying humidity and temperature conditions during almost 1 year prior to this long-term stability experiment, further corroborating the high robustness of this catalytic-plasmonic H_2_ sensor.

**Figure 6:**
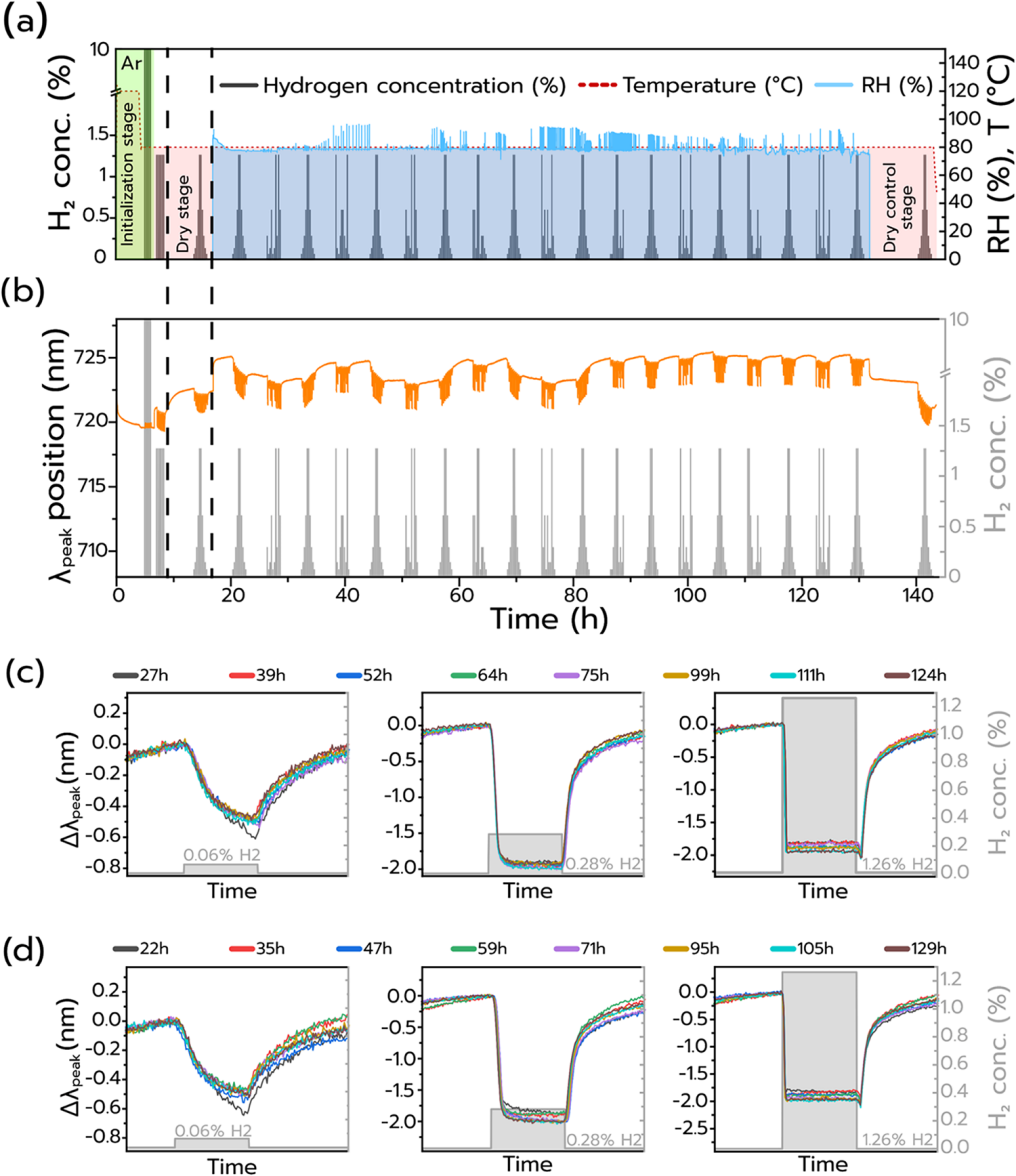
Sensor performance over 143 h in synthetic air, at 80% RH, and 80 °C operation temperature. (a) The gas protocol consists of alternating regular (as in previous measurements) and randomized H_2_ pulse sets, i.e., the same concentration values as in the regular set (0.06–1.26 vol %), but in a random order. The green color denotes the initialization stage where the background gas used is argon, followed by a dry stage where the sensor is exposed to a H_2_ pulse set in 0% RH in synthetic air. The protocol continues with the core section with regular and randomized pulses and ends with the final dry control stage at 0% RH. (b) The obtained optical response of the sensor to H_2_ over time. (c) Overlapping response from selected H_2_ pulses (0.06, 0.28, 1.26 vol %) of regular and (d) randomized pulse sets at different points in time during the long-term stability measurement. For all three H_2_ pulses in both the regular and randomized sets, a consistent and repeatable sensor response is observed.

## Conclusions

In this work, we have designed a Pt nanoparticle array-based catalytic-plasmonic hydrogen sensor and showcased its high performance in high-humidity environments in air. This is enabled by a combination of effects that take place in concert: (i) the susceptibility of the LSPR in Pt nanoparticles toward variations in O- and H-adsorbate-induced diffuse electron scattering and the corresponding change in electron density, which induces an LSPR spectral blue-shift when going from a O-covered surface to a partially H-covered surface, (ii) the high sensitivity of the LSPR in Pt nanoparticles to variations in their closest dielectric environment, which in the context at hand corresponds to adsorbed H_2_O multilayers with humidity-dependent thickness, (iii) the efficient catalytic HOR on Pt nanoparticles that takes place in the presence of H_2_ and O_2_, and whose high exothermicity (proportional to H_2_ concentration when H_2_ is in deficit as in the sensing scenario at hand) leads to desorption of H_2_O layers adsorbed on the surface, and thus a change in the dielectric environment that results in a spectral blue-shift of the LSPR. Taken all together these three effects enabled reliable plasmonic H_2_ detection in high-humidity environments in air, *i.e*., a sensor response to H_2_ within the entire explored humidity range (0–80% RH), where the sensor is able to meet and significantly exceed the DoE target of <1000 ppm of H_2_ LoD at all measured operating temperatures (33–100 °C). Remarkably at the determined optimal operating temperature of 80 °C, the LoD is 50 ppm of H_2_ at the highest explored 80% RH. As a second key observation, we found that the developed catalytic-plasmonic Pt nanoparticle H_2_ sensor exhibited an increasing signal amplitude above a specific H_2_ concentration for increasing humidity levels and for sensor operating temperatures *T* ≥ 50 °C. This feature is in stark contrast to all other reported H_2_ sensing platforms where increasing RH decreases the signal amplitude or even completely deactivates the sensor to the best of our knowledge. Finally, a long-term stability experiment also revealed an overall consistent and constant response to H_2_ in high-humidity conditions of 80% RH for a total of 143 h continuously on stream, and selectivity/deactivation tests in 80% RH in synthetic air involving C_3_H_6_, as well as CH_4_ and CO according to ISO 26142:2010 ^18^, demonstrated high selectivity and deactivation resistance. Taken together, these results demonstrate the potential of combining two sensing principles in one, here implemented as a catalytic-plasmonic H_2_ sensor, to address the technologically highly relevant challenge of detecting H_2_ in highly humid environments.

## Methods

### Sample Fabrication

A quasi-random array of Pt nanodisks with 210 nm nominal (215 ± 10 nm mean*)* diameter and 25 nm height (Figure 1a and Supporting Figure S2a–d) was fabricated using hole-mask colloidal lithography (HCL)^33^ and Pt deposition by electron beam evaporation on a 0.9 × 0.9 × 0.05 cm^3^ fused silica substrate (Siegert Wafer GmbH). For the samples used in the investigation of the effect of surface coverage (SI Section 9), consisting of quasi-random arrays of particles, a 4 in. fused silica wafer (thickness: 500 μm, Siegert Wafer GmbH) was used as substrate. A bilayer resist stack was spin-coated onto the wafer: first 100 nm of MMA(8.5)­MAA (MicroChem Corp.), baked for 5 min at 180 °C on a hot plate, followed by 60 nm of PMMA 950k (MicroChem Corp.), also baked for 5 min at 180 °C. Subsequently, a 20 nm chromium layer was thermally evaporated (Lesker NanoChrome, Kurt J. Lesker Company) onto the resist stack to enable charge dissipation during electron beam lithography and to enhance optical contrast for height metrology. Electron beam lithography was performed by using a Raith EBPG 5200 system at 30 nA beam current with a beam step size of 10 nm and a dose of 500 μC/cm^2^ (with proximity effect correction applied). After exposure, the chromium layer was selectively removed by wet chemical etching for 1 min using Cr-07S (MicroChemicals GmbH). The bilayer resist was then developed in a 4:1 mixture of IPA:H_2_O for 1 min, followed by drying with nitrogen without rinsing. After development, a short oxygen plasma descum was performed to remove residual resist (10 s, 50 W, 50 sccm O_2_) prior to metal deposition. Subsequently, a 25 nm platinum layer was electron beam evaporated (Lesker PVD 225, Kurt J. Lesker Company) at a deposition rate of 2 Å/s. Lift-off was carried out in Remover 1165 (Kayaku Advanced Materials, formerly MicroChem Corp.) for 10 h at room temperature, followed by rinsing in acetone, rinsing in 2-propanol (IPA), rinsing in deionized water, and blow-drying with nitrogen. The wafer was then diced into individual chips. To protect the surface during dicing, a 200 nm layer of PMMA 950k (MicroChem Corp.) was spin-coated prior to processing. Dicing was performed using a DAD3350 dicing saw (DISCO Corporation) equipped with a resin-bonded diamond blade (K015–600JXS, DISCO Corporation) operating at 25 000 rpm spindle speed and a feed rate of 2 mm/s. The protective PMMA layer was then removed by rinsing the samples in acetone, followed by IPA and deionized water, and finally blow-drying with nitrogen.

### Experimental Setup for Sensor Performance, Humidity Titration, and Long-Term Stability Experiments

The setup used for humidity titration measurements, sensor performance measurements, and the long-term stability investigation consists of a quartz tube plug-flow reactor with optical access for transmittance measurements (Insplorion AB). It is equipped with a set of Mass Flow Controllers (MFCs, Bronkhorst High-Tech B.V.) that control the flow rate and gas composition (synthetic air, H_2_), a Liquid Flow Controller (LFC, Bronkhorst High-Tech B.V.) for controlling the water supply rate, and a Controlled Evaporator Mixer (CEM, Bronkhorst High-Tech B.V.) that humidifies the gas. In all measurements, the water supplied from the LFC into the CEM for humidifying the gas stream is referenced to RH values at 30 °C, 1.013 bar, and 200 mL/min total gas flow rate. Specifically for the 3 RH values used in this work, *i.e*., 20, 50, and 80% RH, the corresponding water flow was 0.081, 0.206, and 0.333 g/h, respectively, and was calculated using Bronkhorst’s Fluidat software. The humidity level was measured by a calibrated humidity and temperature probe (HMP7, Vaisala) positioned at the chamber inlet. The reactor temperature was controlled using a closed-loop temperature control system (Eurotherm 3216) in a feedback loop manner, where the sample surface temperature inside the chamber (measured via a K-type thermocouple) was continuously used as the input. The chamber can accommodate up to two samples, which are illuminated using an unpolarized halogen white light source (AvaLight-HAL, Avantes) coupled through a bifurcated optical fiber (FCB-UV600–2, Avantes BV) equipped with collimating lenses. The transmitted light from each sample is collected and analyzed by a dual-channel fiber-coupled fixed-grating spectrometer (AvaSpec-ULS2048CL-2-EVO, Avantes BV).

### Humidity Titration Experiment

The sample was exposed to a constant gas flow rate of 200 mL/min of synthetic air (20.5% O_2_ in N_2_), humidified in increasing/decreasing increments of 10% RH (as described in the previous section) in the range of 0–80% RH (0–0.333 g/h H_2_O flow rate), at four different sensor temperatures (33, 50, 80, 100 °C). Prior to each measurement at a given sensor operation temperature, the sensor was exposed to dry synthetic air for 155 min. Specifically, before the first measurement at 33 °C, the sensor temperature was set to 120 °C under dry synthetic air for 4 h, to ensure complete desorption of adsorbed H_2_O from when the sample was stored at ambient conditions prior to the measurement.

### Sensor Performance Measurements

The measurement gas protocol was divided into: (i) the initialization stage that lasted 4 h, where the sensor temperature was set to 120 °C. During this time, the sensor was exposed to 5 × 10 vol % H_2_ in Ar and 6 × 1.26 vol % H_2_ in synthetic air. The final step of this stage was to reduce the sensor temperature to the temperature of operation (*i.e*., 30, 50, 80, or 100 °C), where the sensor stood idle for 170 min, before the beginning of the next stage. The aim of the initialization stage was to desorb any previously adsorbed H_2_O on the sample’s surface and to acquire a stable sensor baseline. (ii) The core stage that consisted of a constant flow of synthetic air where intermittently H_2_ pulses of increasing and decreasing concentration were introduced. The concentrations were 0.06, 0.13, 0.28, 0.60, and 1.26 vol % H_2_. Within the set, each H_2_ pulse lasted for 300 s, followed by 600 s of 100 vol % of synthetic air gas flow. In total, there were 4 H_2_ pulse sets, one for each humidity background, starting at 0% RH (dry conditions) and followed by sets at 20, 50, and 80% RH. (iii) The control stage: After the highest humidity background (80% RH), the sensor was exposed to a constant synthetic air flow for 11.5 h at 0% RH for each corresponding temperature of operation. After that time, the sensor was exposed to another H_2_ pulse set at 0% RH. To differentiate the two sets at 0% RH (beginning of core stage and the control stage), this one is named “*0% RH after TR”* in Figures 4a,b and[Fig fig5]a, where TR stands for temperature regeneration. In all measurements, the total gas flow rate at any time was set to 200 mL/min.

### Long-Term Stability Investigation

The measurement gas protocol consisted of: (i) the initialization stage, (ii) the dry stage, (iii) the core section, and (iv) the dry control stage. In (i), the sensor’s temperature was set to 120 °C for 4 h. The temperature was then reduced to 80 °C, where the sensor was exposed to 5 × 10 vol % H_2_ in Ar. In (ii), the Ar background gas was replaced by synthetic air, and the sensor was exposed to 6 × 1.26 vol % H_2_ followed by a H_2_ pulse set (as described in the Sensor performance measurements section), in dry conditions (0% RH). In (iii), the background humidity was set to 80% RH and the sensor was exposed to a total of 19 H_2_ pulse sets, alternating between regular and randomized sets. The regular H_2_ pulse sets were identical to that in the Sensor Performance Measurements section, while the randomized ones contained the same H_2_ concentrations but in random order. This section lasted for 114 h, simulating the sensor performance under high-humidity conditions for an extended period of time. Finally, in (iv), the background humidity was reduced to 0% RH, and after 8 h, the sensor was exposed to a final regular H_2_ pulse set. The protocol was 143 h in total, and the total gas flow rate at any time was set to 200 mL/min. In Figure 6b, where we overlap H_2_ for 3 different H_2_ concentrations within the regular pulse sets, each concentration was used twice. Specifically for the 0.06 vol % H_2_, that pulse was present at the beginning and the end of the set.

### Quadruple Mass Spectrometry (QMS) Experiment

The QMS experiment was carried out in an externally heated quartz tube plug-flow reactor with an integrated glass pocket that we have reported earlier^59^ and as conceptually introduced by Bu et al.^60^ The gas composition and flow rate were controlled by a series of MFCs (Bronkhorst High-Tech B.V.). The sample was placed in the glass pocket, which serves to minimize the dilution of reaction products, and it is further connected to a differentially pumped quadruple mass spectrometer (Pfeifer, OmniStar GSD320). In the absence of H_2_, the sample was exposed to a constant flow of dry synthetic air (200 mL/min). Intermittently, 4 different H_2_ pulses were introduced in the reactor, *i.e*., 0.13, 0.28, 0.60, and 1.26 vol % for all 4 different sensor temperatures (33, 50, 80, 100 °C). The total flow rate was kept constant at 200 mL/min. The QMS was operated in selected ion monitoring (SIM) mode, where the ion current specific to H_2_O ions was monitored.

### Electron Microscopy

Scanning electron microscopy (SEM) imaging (Figure 1a and Supporting Figure S2a–d) was carried out in a Zeiss GeminiSEM 450 microscope, using a Pt sample that was fabricated on a Si substrate to mitigate charge buildup (using the same fabrication method as described previously). All images were collected with a secondary electron detector, and the acceleration voltage was 10–15 kV. For the samples used in the surface coverage investigation (Supporting Figure S11a–d), an acceleration voltage of 2 kV and 10pA current were used for imaging, due to the nonconductive substrate. The transmission electron microscopy (TEM) images were taken using an FEI Tecnai T20 microscope operated at 200 kV with a LaB6 filament. The microscope is equipped with an Orius CCD camera. The particles were fabricated onto electron-transparent silicon nitride membranes that allows for seamless localization and imaging of the same particles at separate microscopy sessions.

## Supplementary Material



## Notes

Introduction to nanoplasmonic and catalytic sensors, TEM/SEM imaging of Pt nanodisks, FDTD simulations, assessment of the HOR on the Pt sensor surface, sensor performance measurements at intermediate RH levels, LoD investigation in humid conditions, investigation of the effect of surface coverage variation to the response of the sensor, response/recovery times, selectivity/poisoning resistance to interfering gases, baseline drift in the long-term stability measurement, Pt/Pd comparison, and comparison of Pt/Pd-based H_2_ sensors in humid environments (PDF)
