# Supplementary material for: A Catalytic-Plasmonic Pt Nanoparticle Sensor for Hydrogen Detection in High-Humidity Environments
Source: ACS Sens. 2025 Nov 18;10(11):8983–94. doi: 10.1021/acssensors.5c03166 (PMC12670998; doi:10.1021/acssensors.5c03166)
Supplement: Supplementary file 1 [file se5c03166_si_001.pdf]

Supporting Information

for

A Catalytic-Plasmonic Pt Nanoparticle Sensor for Hydrogen Detection in High Humidity Environments

Athanasios Theodoridis[1], Carl Andersson[1], Sara Nilsson[1], Joachim Fritzsche[1] and Christoph Langhammer[1]*

[1]Department of Physics, Chalmers University of Technology; SE-412 96 Gothenburg, Sweden

*Corresponding author: clangham@chalmers.se

Section 1: Nanoplasmonic hydrogen and dielectric sensors

State of the art plasmonic H_2 sensors rely on the ability of nanoparticles made from Pd and its alloys to both barrierlessly dissociate H_2, and subsequently absorb hydrogen into solid solution and hydride phases.[1] These hydrogen sorption processes induce changes in the electronic structure of the particles, as well as lead to sizable volume expansion, which in concert induce a frequency shift of the localized surface plasmon resonance (LSPR) that is proportional to the H_2 concentration surrounding the particles.[2-5] Employing this concept, high-performance H_2 sensors with respect to multiple of the aforementioned performance targets have been demonstrated.[1] However, in humid environments Pd-based sensors readily deactivate[6] and only recently, employing neural network-based data treatment, we have been able to demonstrate a PdAu alloy nanoplasmonic H_2 sensor with a LoD of 100 ppm at 80 % RH.[7]

An even older and more widely established application of plasmonic sensors is so called dielectric sensing first applied in nanoplasmonic biosensing. Here, the ability of plasmonic nanoparticles to detect minute changes in the dielectric properties of their closest environment, induced for example by the adsorption or specific binding of a biomolecular analyte and again manifested as a frequency shift of the LSPR, is exploited.[8, 9] Mechanistically, this so-called dielectric sensing can be understood as that a perturbation of the refractive index (RI) of the medium localized within the enhanced near-field of the plasmonic particle excited at resonance is induced by the adsorption of (single) molecules[10] or films in the Ångström to nanometer thickness range.[8] The measured spectral shifts of the resonance peak are proportional to the number of adsorbed molecules or the thickness of the film within certain limits.[8, 11] From a technical perspective, plasmonic sensors have already been commercialized[12, 13] and rely on simplified but highly efficient (single-wavelength) readout schemes, which significantly simplify field deployment.

Section 2: Catalytic and resistivity-based Pt hydrogen sensors

On Pt-group metal surfaces the HOR can take place, and Pt surfaces in particular constitute a highly active catalyst for the HOR. On such Pt surfaces, dissociated chemisorbed oxygen[14] reacts with dissociated chemisorbed hydrogen and forms water that thermally desorbs to the gas phase,[15] according to

$$O_2 \rightarrow 2O_{ads}\ (dissociation)$$

$$H_2 \rightarrow 2H_{ads}\ (dissociation)$$

$$2H_{ads} + O_{ads} \rightarrow H_2O\ (desorption)$$

This reaction is highly exothermic (-241.8 kJ/mol)[16] and has been demonstrated to occur efficiently already at ambient temperature on polycrystalline Pt nanoparticles.[15, 17]

In the (H_2) gas sensor context, catalytic sensing is widely established and harnesses the chemical heat generated in catalytic combustion processes of analytes, such as H_2, as the readout. Such sensors are typically of the Pellistor-type,[18] where a so-called Wheatstone bridge is used to measure a temperature-increase-induced change in electrical resistance that is proportional to the H_2 concentration.[19, 20] An alternative design is the thermoelectric-type, based on the Seebeck effect, where a difference in temperature between two points leads to a voltage difference that can be correlated to the change in H_2 concentration.[21] Such catalytic H_2 sensors can be elegantly miniaturized, exhibit low LoDs and reasonably fast response times, but they readily deactivate in high humidity environments.

A second type of hydrogen sensors for ambient environments in air where Pt is used as the sensing material are resistivity sensors. In these sensors, a difference in adsorbate-induced diffusive electron scattering between O- and H-covered surfaces provides the detection mechanism *via* corresponding changes in the Pt resistivity, which conveniently can be measured.[22-24] Specifically, at higher O-coverage of the Pt surface the diffusive electron scattering increases due to the high electronegativity of O and the corresponding "withdrawal" of electrons from the Pt surface to the chemisorbed O. Consequently, the electrical resistance of the material also increases. In contrast, upon H_2 exposure, depending on the partial pressure, a certain fraction of O will be replaced by dissociated chemisorbed H, which reduces the diffusive electron scattering since H is electropositive, and thus reduces the electrical resistance relative to an O-terminated surface.[22, 23] This relative change is used as the sensing mechanism in resistive Pt sensors operated in an O_2 rich environment, such as ambient air. Examples of such sensors include Pt nanowires, either single[22] or as an array,[23] where in the first case a single platinum nanowire has been shown to achieve a 10 ppm LoD with a response time of ~150 s at 0.1 vol.% H_2 in air, however at significantly elevated operating temperature (T=550K).[22] In the latter case, the H_2 sensing performance of a dense array of Pt nanowires has been investigated, showing a remarkable LoD of 1 ppm at 298K, in air.[23] It is important to note that both studies were conducted in dry air, and that the performance of such sensors in humid conditions has not been addressed to-date. Contrary to these studies, Cao *et al.*,[24] reported on defect-rich Pt nanowires, fabricated by focused ion beam, where H_2 induces a resistance *increase* upon adsorption and dissociation, a feature that is attributed to defect-dominated electron scattering, as opposed to the difference in the surface inelastic electron scattering between O and H adsorbed atoms, which causes a decrease in resistance upon exposure to H_2. The sensor exhibits a remarkable LoD of 10 ppb in either N_2 or dry air (not specified), and the effect of H_2O is also addressed (albeit only at 15 and 50% RH), showing a negative influence of environmental humidity in the sensor's response magnitude. As another example, Lim et al.[25] developed a Pt/LIG (laser-induced graphene) structure, that is capable of reaching an outstanding LoD

of 200 ppb in dry air and room temperature. However, the reported sensor has only been subjected to 1 vol.% H_2 in the 0-75% RH range, showing a decrease in the sensor's response magnitude, with increasing humidity. Finally, Pt has also been used in decorating Pd[26] and PdO[27] nanowires, with overall good performance at room temperature, such as LoDs in the low ppm regime. However, none of these studies have investigated the sensor's performance in humid conditions.

Section 3: TEM/SEM imaging

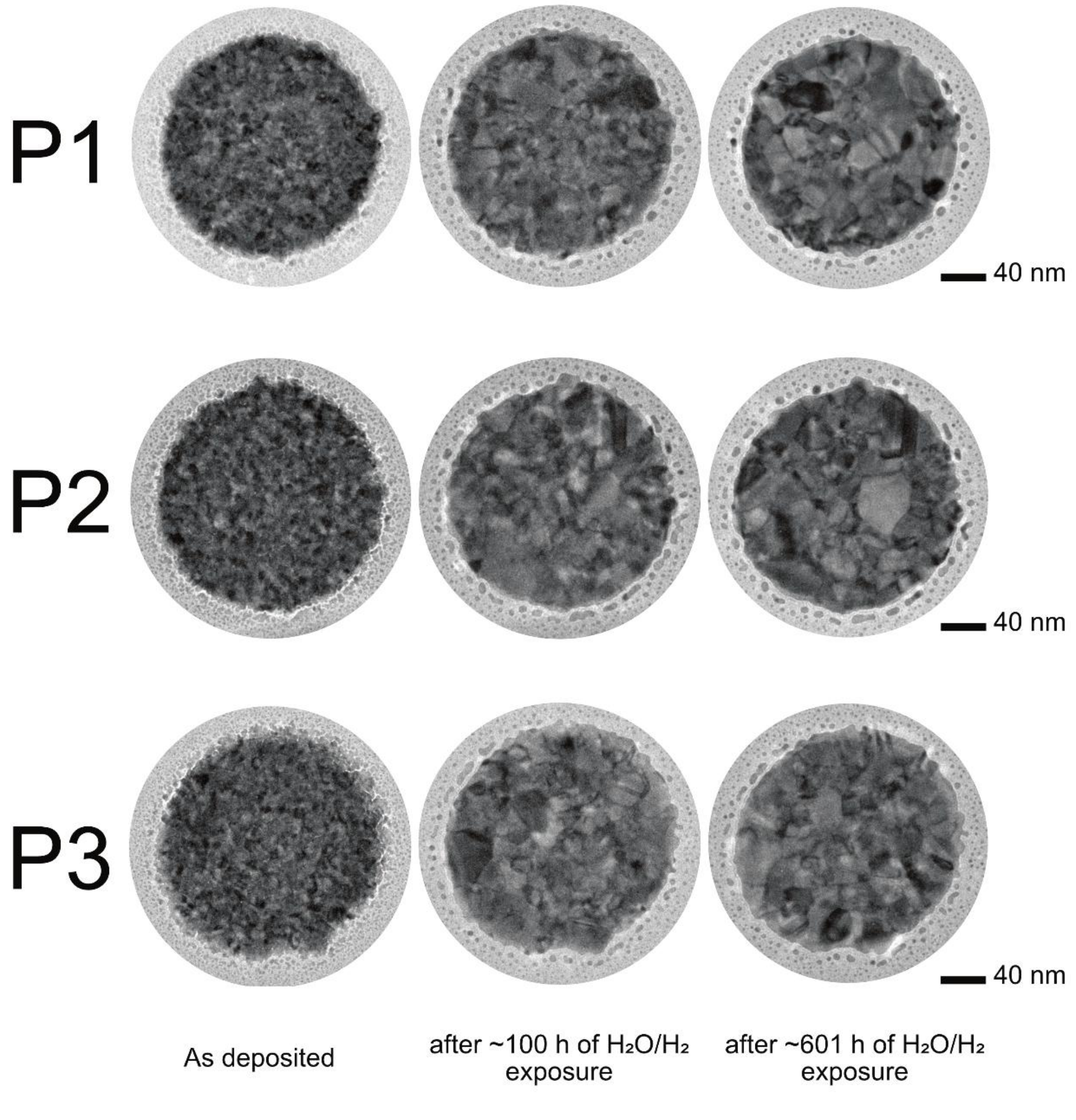

Figure S1: *Bright-field TEM images of 3 different particles named P1, P2, and P3. For each particle, three images were taken at 3 different times: (i) directly after nanofabrication (as deposited) and without any exposure to H_2 or humidity, (ii) after ~100 and (iii) after ~601 hours of exposure at varying conditions which include humidities in the 0-80% range, and H_2 pulses of varying concentrations (0.06*

– 10 vol.%). In the as-deposited state, the particle is characterized by high polycrystallinity, typical for materials deposited via physical vapor deposition. Upon exposure to H_2O/H_2, the particles undergo a recrystallization process, as consequence of the exothermic hydrogen oxidation reaction (HOR). Notably, despite this recrystallization, the overall size and shape of the disk-like particle remains completely unchanged. The orientation for each set of particle images is the same.

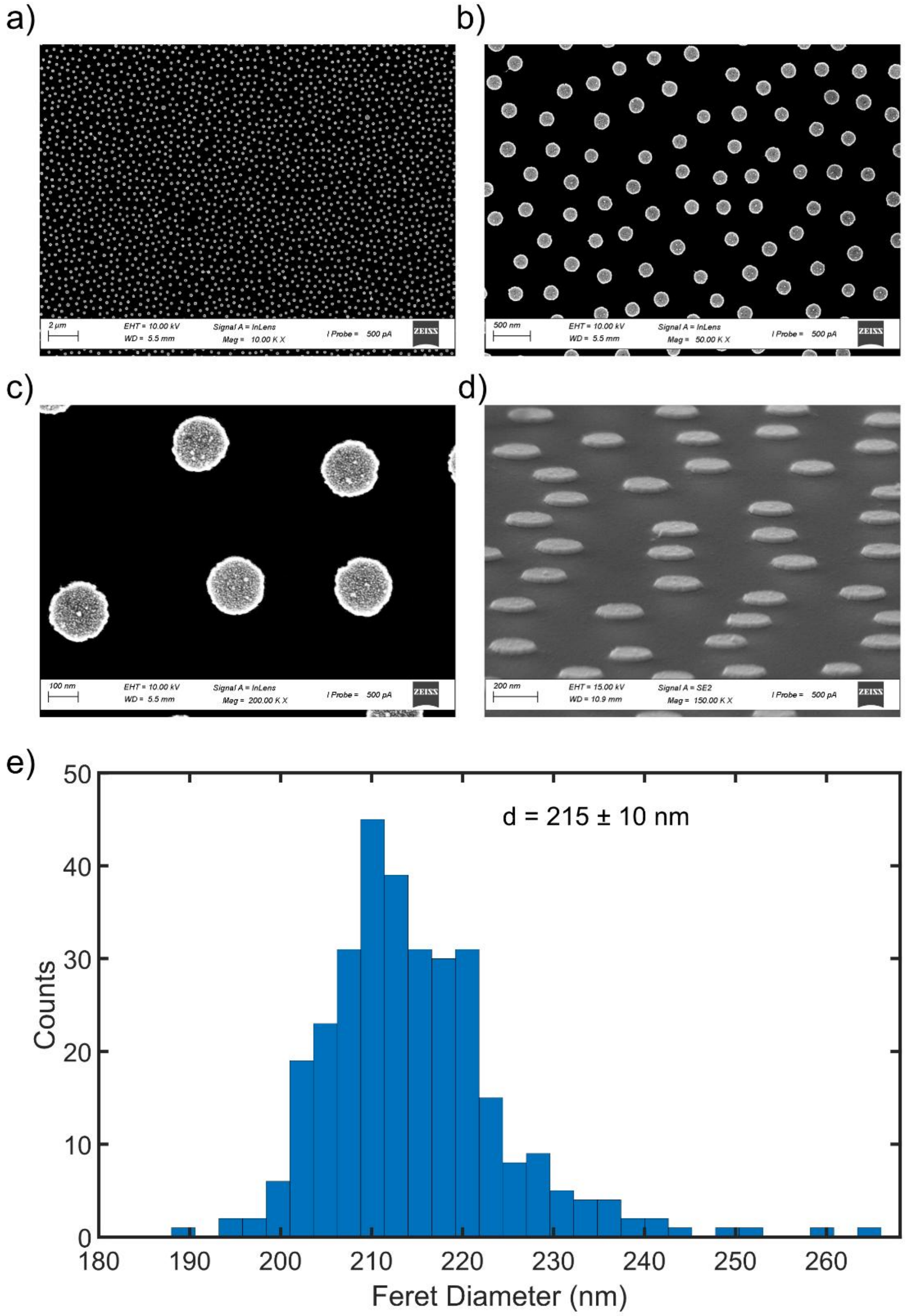

Figure S2: *Top view SEM images of the Pt nanodisks at different magnifications (a-c). d) Side view of*

the particles at 45° tilt and e) histogram of the Feret diameter obtained from 314 imaged Pt nanodisks, collected from top view SEM images. The mean diameter was 215 ± 10 nm. The software used to measure the disk sizes was ImageJ.

Section 4: FDTD Simulations

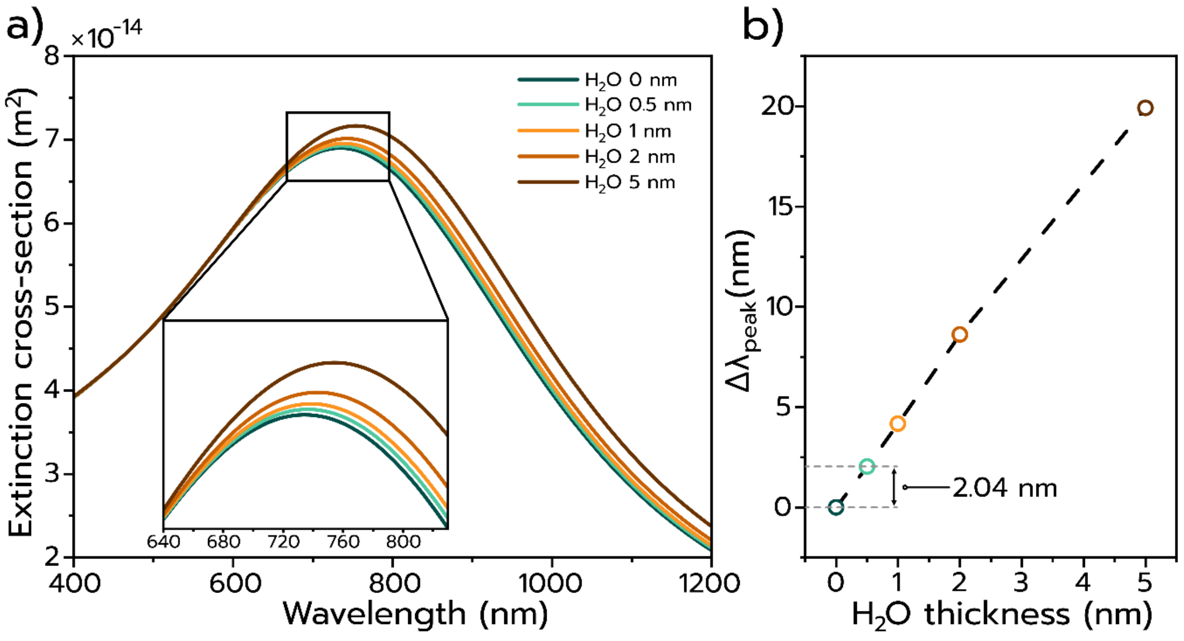

Figure S3: *a) FDTD simulations of the extinction cross-section for a Pt nanodisk with a diameter of 215 nm and 25 nm height (same as the dimensions for the fabricated nanodisks), with adsorbed water layers of different thickness. b) The peak shift extracted from a) for the different water layer thicknesses. A 0.5 nm thick water layer yields a 2.04 nm shift of the peak compared to a completely dry surface.*

A rounded Pt disk, with diameter 215 nm, height 25 nm and tapering 20° to emulate the nanofabricated nanodisks used in the experiment, was simulated on a silica support using the finite-difference time-domain (FDTD) method in ANSYS Lumerical FDTD. A thin layer with refractive index n = 1.33 (equal to the RI of water) was added around the particle to emulate the layers of adsorbed water. The incoming electrical field was simulated as a plane wave using a total-field/scattered-field source. The scattering and absorption cross-sections were obtained by integrating the Poynting vector of the scattered and absorbed (the difference between incoming and scattered fields) fields, respectively. The scattering and absorption cross-sections were summed to obtain the extinction cross-sections depicted in **Figure S3a**. In **Figure S3b**, the λ_{peak} for each spectrum was calculated *via* a centroid fitting algorithm, where the peak was fitted to a high-degree polynomial (20th order).[11]

Section 5: Assessing the HOR on the Pt sensor surface

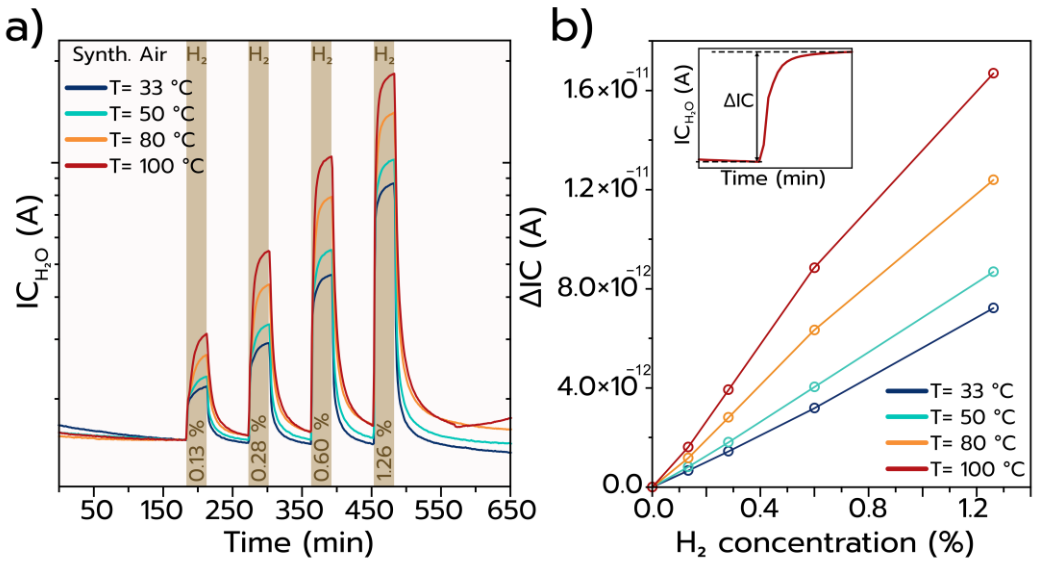

Figure S4: *Mass spectrometry of the HOR on the Pt sensor surface. (a) The H_2O ion current (IC) measured by the QMS over time for a Pt sensor surface exposed to a constant synthetic air background and 30 min H_2 pulses at 4 different temperatures indicated in the figure. (b) Ion current difference (ΔIC – referenced to the Pt sensor exposed to synthetic air only) measured for the four different H_2 concentrations at the four different temperatures in (a). It reveals the expected temperature dependence of the catalytic rate, as well as the first order kinetics of the HOR expected for the low H_2 concentrations at hand here, with O_2 always available in excess. The inset depicts how ΔIC is calculated.*

To assess the HOR catalytic activity of the Pt sensor surface, we employed quadruple mass spectrometry (QMS) at the same four temperatures as used above for humidity titration (***c.f* Figure 2**). For this purpose we used an externally heated quartz-tube plug-flow reactor with an integrated glass pocket,[28] as conceptually introduced by Bu *et al.*.[29] This pocket minimizes dilution of reaction products from nanofabricated samples, like the Pt sensor surface at hand here, and thereby enables QMS analysis of reaction products (see **Methods** for details). The reactor was constantly flushed with synthetic air at 200 ml/min flow rate, and intermittently exposed to H_2 pulses of 0.13, 0.28, 0.60 and 1.26 vol.%, respectively, while tracking the QMS ion current response for H_2O, IC_{H2O} (**Figure S4a**). Evidently, H_2O is formed during the H_2 pulses and desorbs from the Pt sensor surface. As expected, the rate of H_2O formation by the HOR is both proportional to H_2 concentration (since O_2 is in excess) and temperature. Further analyzing the change in H_2O ion current, ΔIC, as function of H_2 concentration reveals first order kinetics, as expected at the low H_2 concentrations at hand here (**Figure S4b**).

Section 6: Intermediate RH measurements & low H_2 concentration zoom-in figure

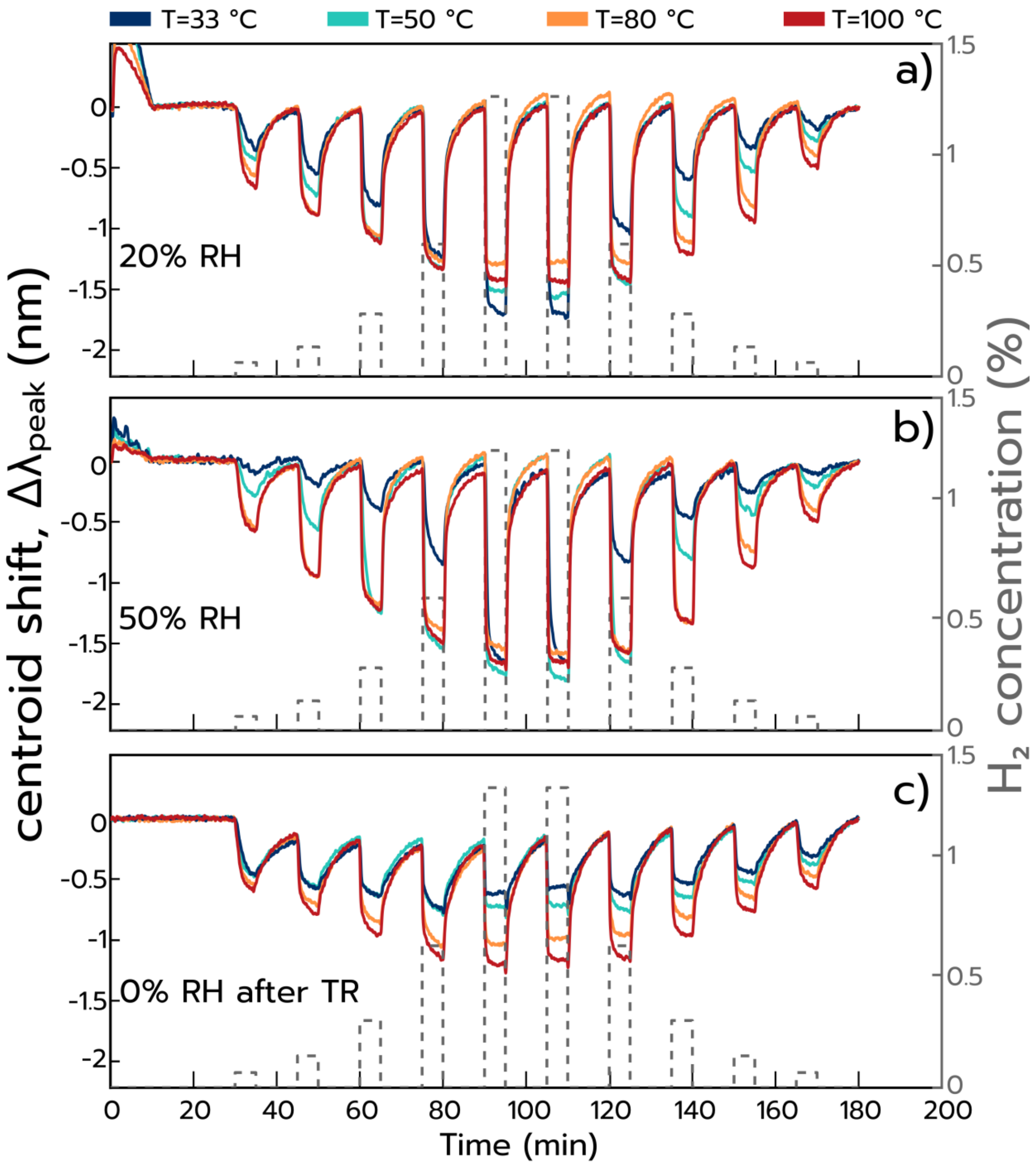

Figure S5: *Sensor performance over time at 33, 50, 80, and 100 ºC for a) 20% RH, b) 50% RH and c) 0% RH after TR, were "TR" = Temperature Regeneration. The left y-axis shows the shift of the centroid, normalized to the value at the beginning of each set of pulses. The right y-axis shows the hydrogen concentrations ranging from 0.06 to 1.26 vol.%.*

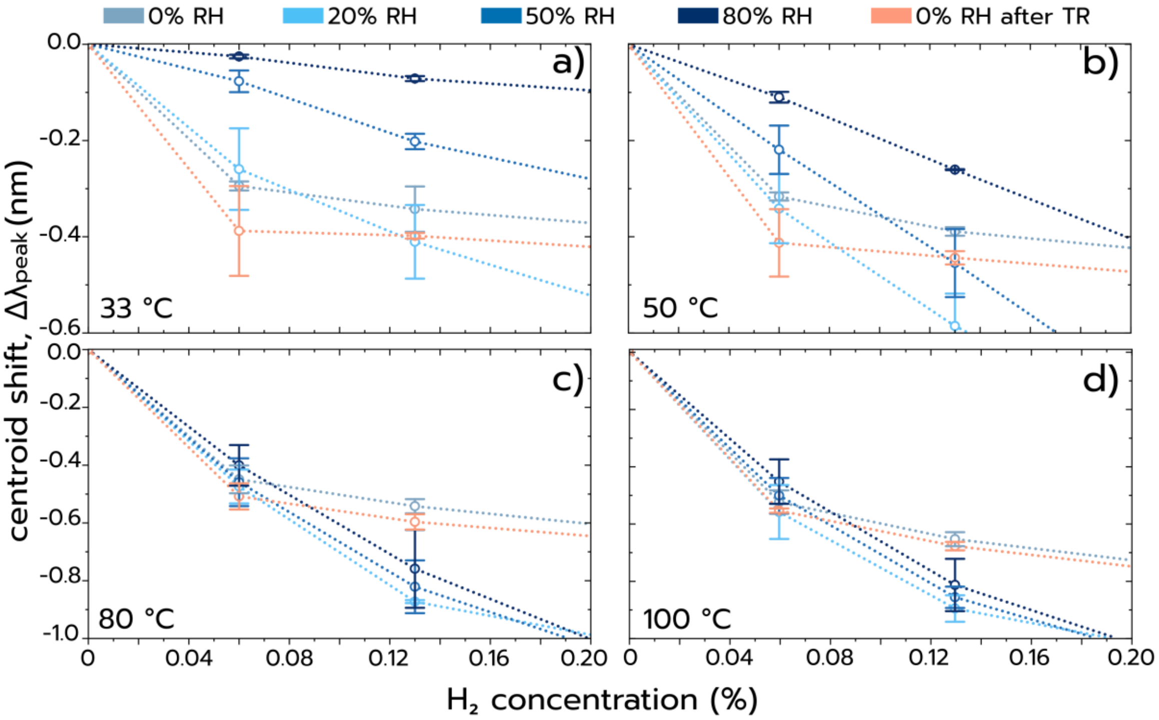

Figure S6: *Zoomed in figure at the 0-0.2 vol.% H_2 concentration range, adjusted from* ***Figure 5*** *in the main text. The centroid shift, $\Delta\lambda_{peak}$, as function of H_2 concentration at a) 33 ˚C, b) 50 ˚C, c) 80 ˚C, and d) 100 ˚C. Each panel depicts 5 curves corresponding to 0% RH (at the beginning and after TR), 20% RH, 50% RH and 80% RH. Each point corresponds to the average between 2 identical H_2 pulses within the same pulse set, where the error bar corresponds to the difference between them.*

Section 7: Sensing measurements in expanded H_2 concentration range down to 30 ppm

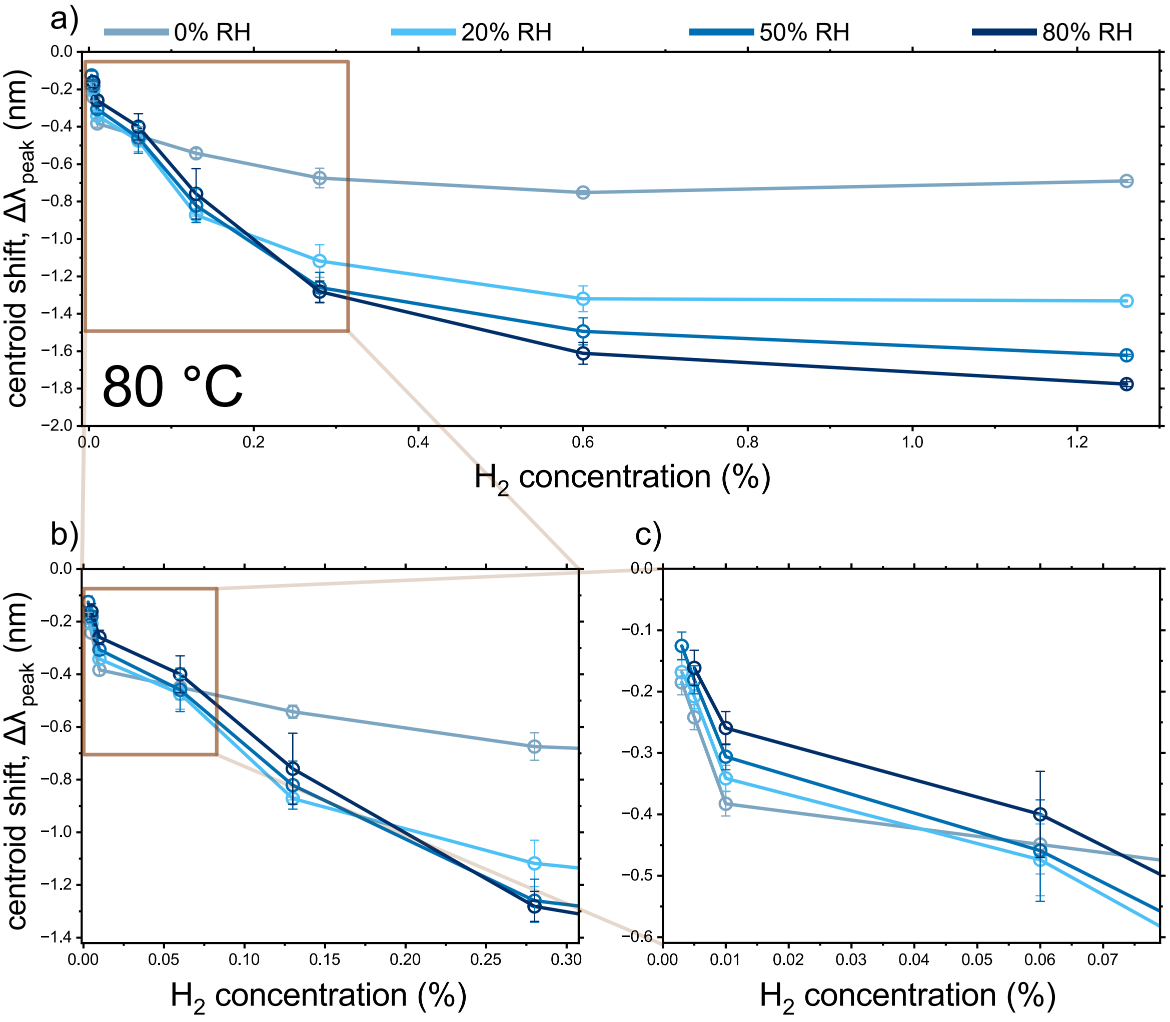

Figure S7: *a) The centroid shift ($\Delta\lambda_{peak}$) as function of H_2 concentration for an extended concentration range of 0.003 – 1.26 vol.% H_2 at the identified optimal sensor operation temperature of 80 ºC. Note that the data points from 0.06 vol.% and above are the same as in* ***figure 5c*** *of the main text. b) Zoom-in to the H_2 concentration region below 0.3 vol.%. c) Further zoom-in to the concentration region below 0.08 vol.% H_2.*

To identify the true LoD of this sensor, we have conducted separate measurements in the low H_2 concentration regime, down to 0.003 vol.% or 30 ppm (see **Methods,** *Experimental Setup for sensor performance, humidity titration and long-term stability experiments* in the main text). **Figure S7** shows the response of the sensor for the extended H_2 concentration range of 0.003 -1.26 vol.%, at 4 different RH (0, 20, 50, 80%) for the identified optimal sensor operation temperature of 80 ºC. The plot is a combination of the initial sensing performance measurement (0.06 – 1.26 vol.%, ***cf.*** **Figure 5** in the main text) and the additional LoD measurement (0.003 – 0.01 vol.%). As the H_2 concentration decreases, below a certain threshold, the amount of H_2 is so low that the HOR is very limited and thus

can no longer desorb a sufficient amount of water from the surface to induce a corresponding plasmonic signal. As a result, in humid conditions, the performance of the sensor deteriorates, with increasing humidity and decreasing concentration, as opposed to the dry conditions. Looking closely at the response at 0.13 vol.% H_2 **(Figure S7b)**, the following effect can be observed; At 20% RH the $\Delta\lambda_{peak}$ is indeed larger than at 0% RH, showcasing that the amount of H_2 supplied is enough to induce thermal desorption of water on the surface, and therefore an additional blue-shift of the plasmon peak, as described in the main text. As the humidity increases (*i.e.* 50 and 80% RH) the $\Delta\lambda_{peak}$ becomes smaller, but still larger than in dry conditions (0% RH). As the H_2 concentration decreases, and looking at the 0.06 vol.% H_2 pulse **(Figure S7c)**, the $\Delta\lambda_{peak}$ for the 80% RH is smaller than that of the dry conditions, indicating that the increased thickness of the adsorbed H_2O, combined with the decreased availability of H_2 leads to a limited amount of HORs taking place at the surface of Pt, and therefore a temperature increase that is insufficient for efficient thermal desorption of H_2O. As the concentration further decreases, the adsorbed H_2O hinders the ability of (the even more limited amount of) H_2 to reach the surface, therefore causing a negative effect on both sensing mechanisms, *i.e.*, electron density change (dominant in dry conditions) and dielectric plasmonic mechanism (present at higher H_2 concentrations where the thermal desorption of H_2O leads to change in the refractive index of the medium surrounding the Pt particles).

Section 8: Limit of Detection (LoD)

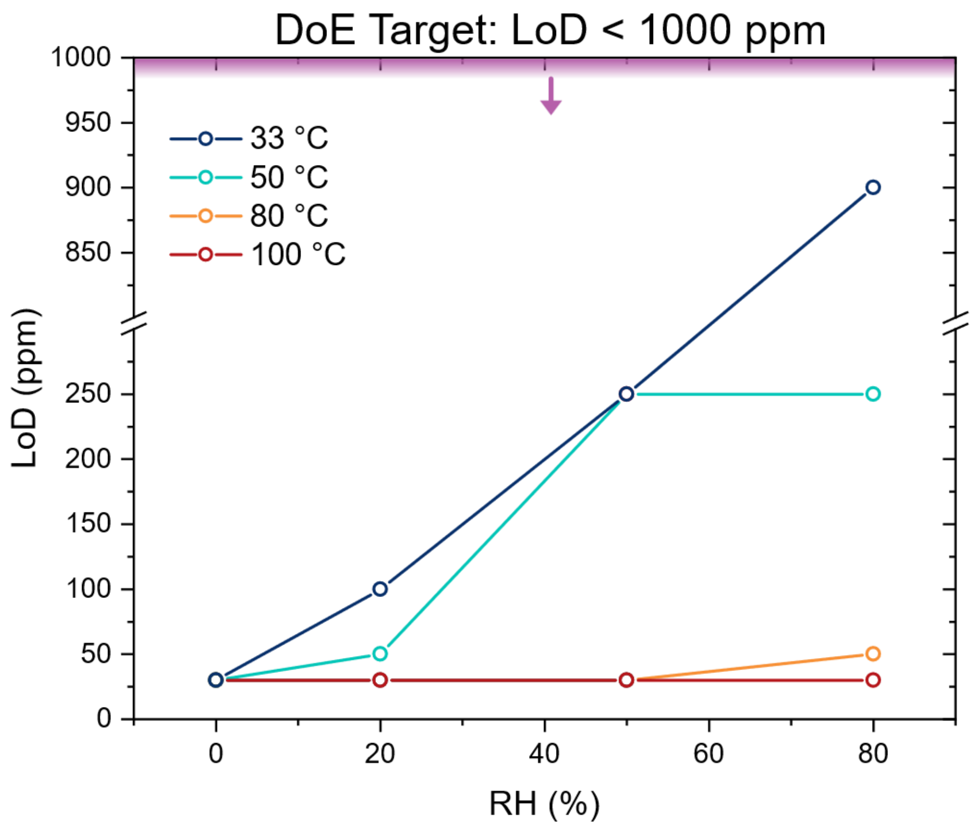

Figure S8: *The LoD as measured for different levels of RH at 33 ºC, 50 ºC, 80 ºC, 100 ºC. The LoD here is defined as the smallest measured H_2 concentration for which the $\Delta\lambda_{peak}$ is larger than 3 times the standard deviation (3σ) of the baseline signal.*

Figure S8 shows the measured LoD for the four tested sensor operation temperatures 33 ºC, 50 ºC, 80 ºC, 100 ºC at different RH. The LoD is defined as the smallest measured H_2 concentration for which the $\Delta\lambda_{peak}$ is larger than 3 times the standard deviation (3σ) of the baseline signal (99.7% confidence). As is evident, all temperatures of operation meet or significantly exceed the DoE target of 1000 ppm for the entire humidity range. More specifically for 80 ºC (optimal temperature of operation as described in the main text) the LoD is more than one order of magnitude below the DoE target, *i.e.*, we measure an LoD of 30 ppm for 0-50% RH and of 50 ppm for 80% RH. **Figures S9, S10** shows the response of the sensor at each H_2 pulse defined as the LoD, alongside the ±3σ.

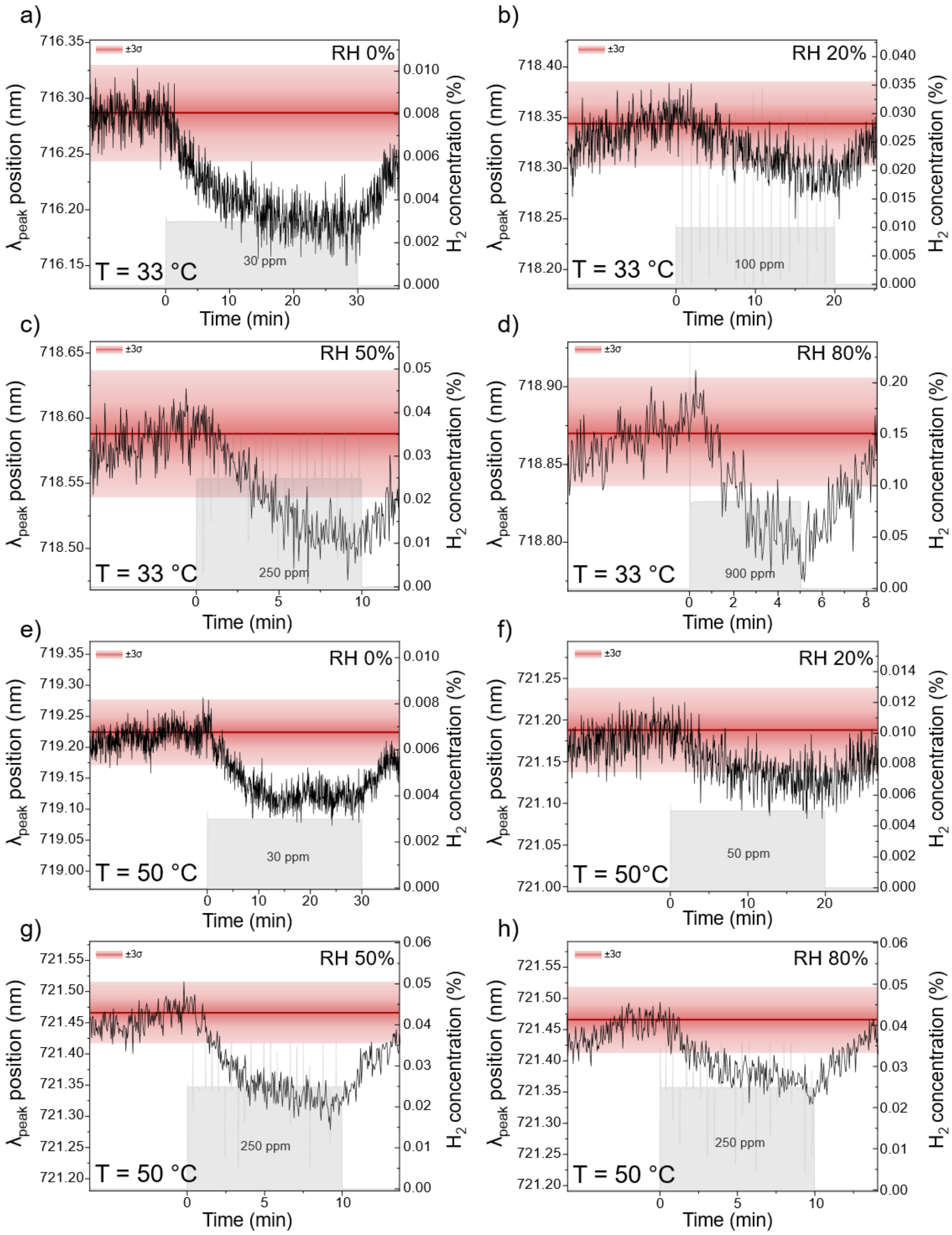

Figure S9: *The position of the peak over time, during a H_2 pulse. Each selected pulse (which determines the LoD at a specific temperature & RH) corresponds to the concentration for which the response of the sensor at the end of the pulse is larger than 3σ of the baseline signal before the pulse. The red line denotes the mean value of the baseline signal before H_2, and the gradient red box denotes the 3σ. Panels a-d) correspond to T = 33 ºC, and RH 0, 20, 50, 80%. Panels e-h) correspond to T = 50 ºC, and RH 0, 20, 50, 80%.*

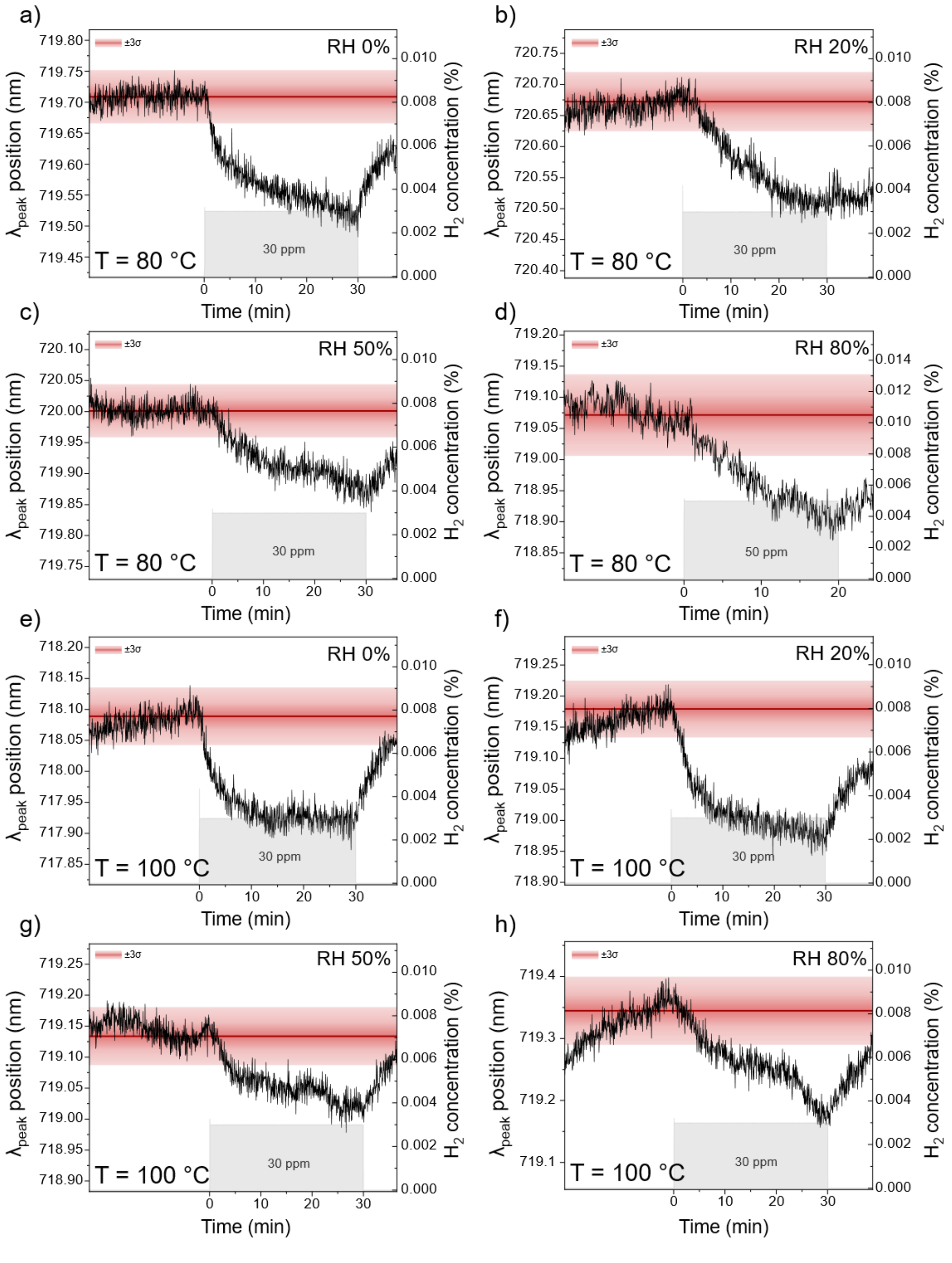

Figure S10: *The position of the peak over time, during a H_2 pulse. Each selected pulse (which determines the LoD at a specific temperature & RH) corresponds to the concentration for which the response of the sensor at the end of the pulse is larger than 3σ of the baseline signal before the pulse. The red line denotes the mean value of the baseline signal before H_2, and the gradient red box denotes the 3σ. Panels a-d) correspond to T = 80 ºC, and RH 0, 20, 50, 80%. Panels e-h) correspond to T = 100 ºC, and RH 0, 20, 50, 80%.*

Section 9: Pt nanodisk surface coverage variation

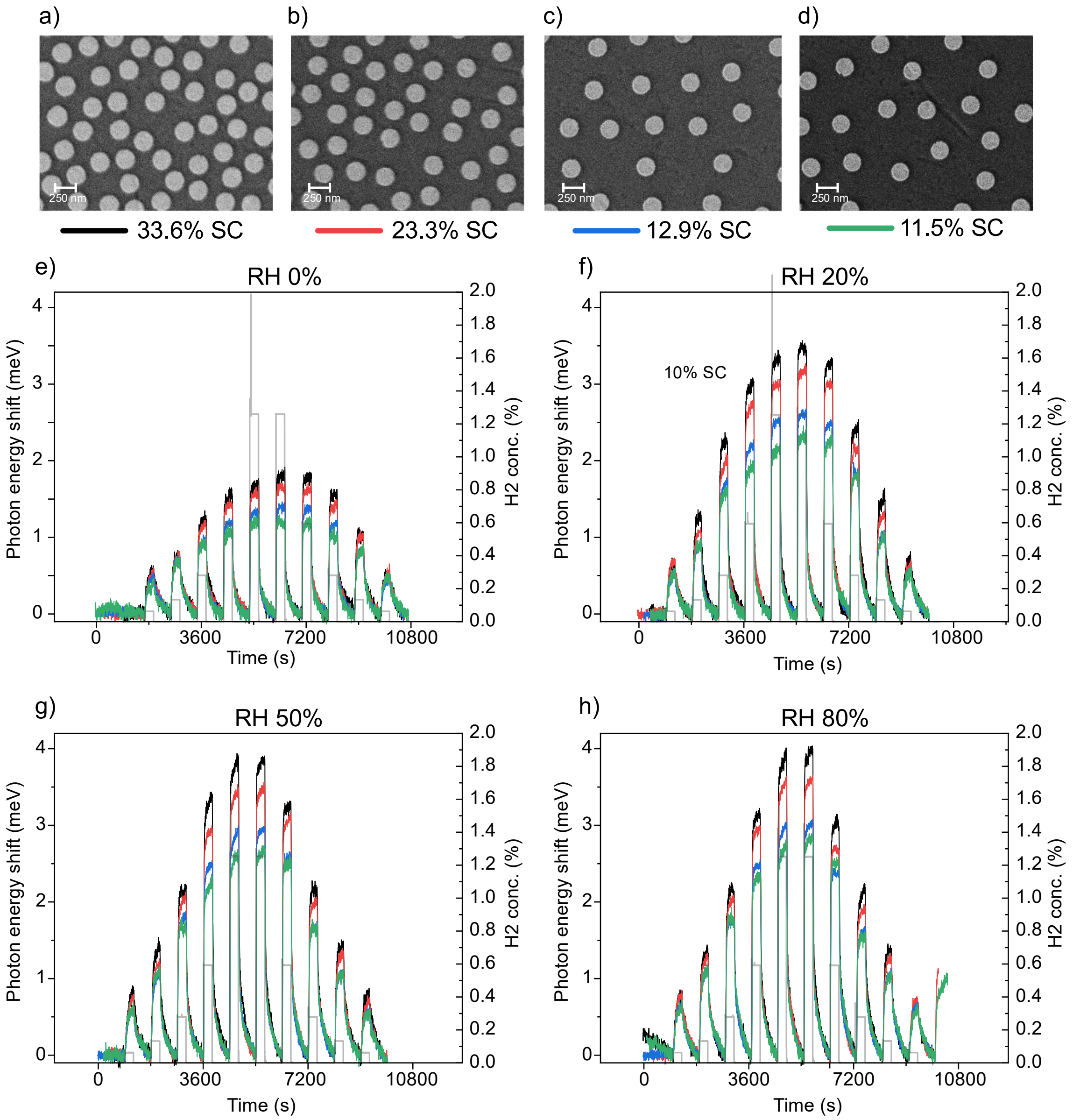

Figure S11: *Baseline corrected photon energy shift as function of time, measured at 80 °C during a set of H_2 pulses consisting of 0.06, 0.13, 0.28, 0.60, 1.26 vol.% H_2, for 4 samples with 33.6 ± 0.9, 23.3 ± 0.5, 12.9 ± 0.7, 11.5 ± 0.3 % Pt nanodisk surface coverage (SC). a-d) Top view SEM images of the 4 samples. The ImageJ software was used to determine the corresponding surface coverages. e) 0% RH, f) 20% RH, g) 50% RH and h) 80% RH. We note the Pt nanodisk surface coverage dependence of the peak shift, which indicates more significant water desorption due to the HOR at higher Pt particle surface coverages due a larger amount of active sites per surface area and possibly also a collective heating effect.*[30] *We also note that the surface coverage dependence in "dry" conditions is the*

consequence of the fact that there is residual water in the gases we use and thus always a thin water layer, as also elaborated in the main text.

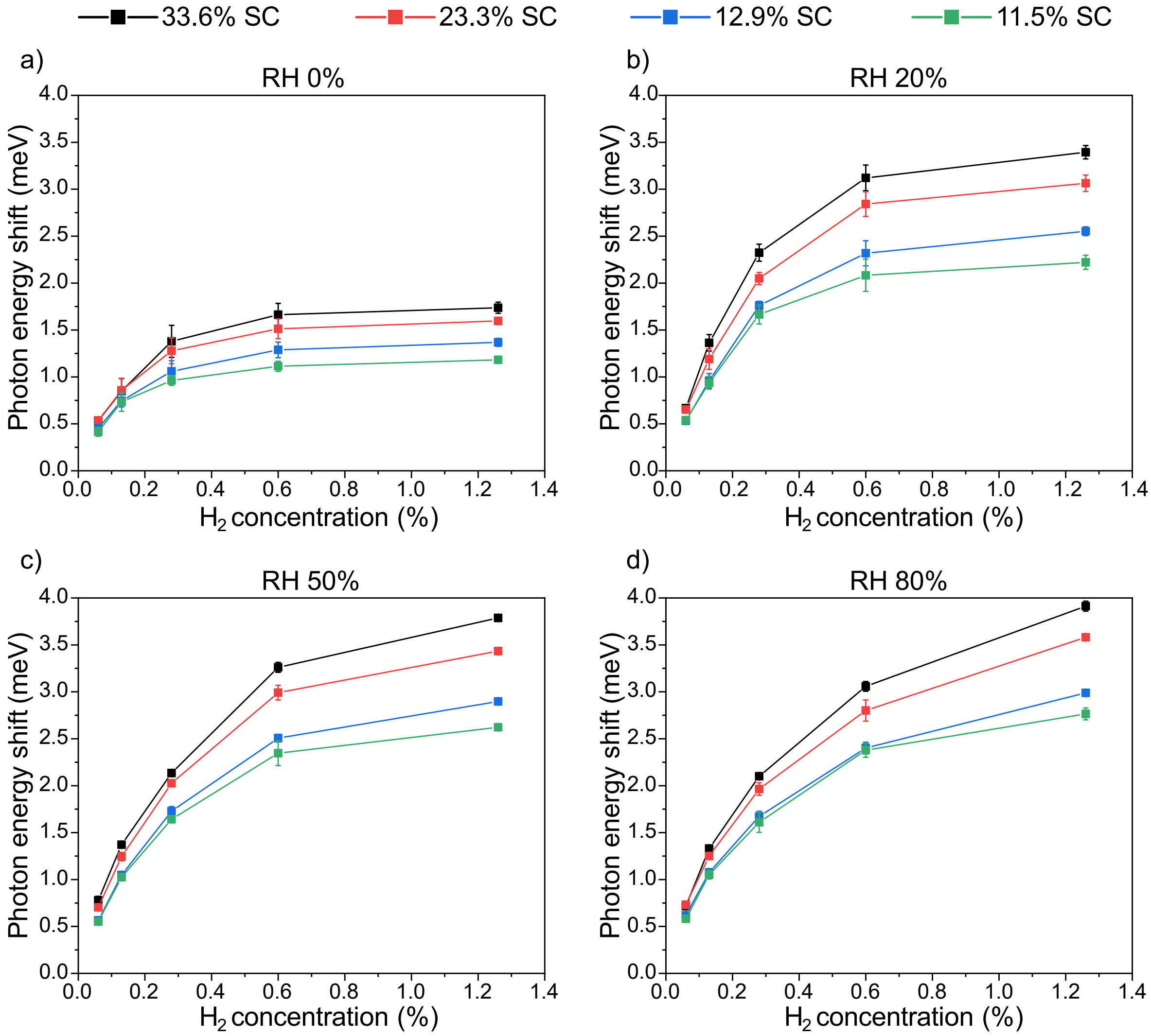

Figure S12: *Photon energy shift of the sensors as function of H_2 concentration for 4 samples with 33.6 ± 0.9, 23.3 ± 0.5, 12.9 ± 0.7, 11.5 ± 0.3 % Pt nanodisk surface coverage, measured at T = 80 °C. a) 0% RH, b) 20% RH, c) 50% RH and d) 80% RH. We note that the surface coverage dependence in "dry" conditions is the consequence of the fact that there is residual water in the gases we use and thus always a thin water layer, as also elaborated in the main text.*

To further corroborate the suggested mechanism of H_2O thermal desorption due to the HOR, we have fabricated Pt sensor samples with varying Pt nanodisk surface coverage, in a quasi-random array to emulate the HCL process, using electron-beam lithography (see **Methods**, *Sample fabrication* for more details). We also note here that the spectral position of the LSPR peak is affected by the Pt nanodisk surface coverage due to near-field effects.[31] Hence, it is expected that the λ_{peak} shift for a *constant*

amount of desorbed water will be different for different surface coverages since the sensitivity towards this desorption, and thus the induced λ_{peak}, is proportional to the LSPR wavelength.[32] To mitigate this effect, we convert the measured spectra from wavelength to photon energy, and hence extract photon energy shifts of the LSPR, rather than the wavelength shift of λ_{peak}, in the analysis of the sensor response as function of Pt nanodisk surface coverage. **Figure S11** and **Figure S12** depict the corresponding photon energy shifts as a function of time when the sensors are exposed to pulses of different H_2 concentrations in the range of 0.06 – 1.26 vol.% for 4 different samples with Pt nanodisk surface coverages indicated in the figure, and measured at 80 °C and four different RH. As shown in all panels, the response magnitude for all H_2 concentrations is increasing with increasing surface coverage, as expected due to a stronger heating effect induced by the HOR, leading to a more efficient thermal desorption of adsorbed H_2O layers. Note that this effect is also present at 0% RH. As described in the main text, this stems from the fact that even in dry conditions, a small amount of water is present, due to traces amounts of H_2O in the supplied gas and diffusion of small amounts of ambient air into the reactor.

Section 10: Response & recovery times

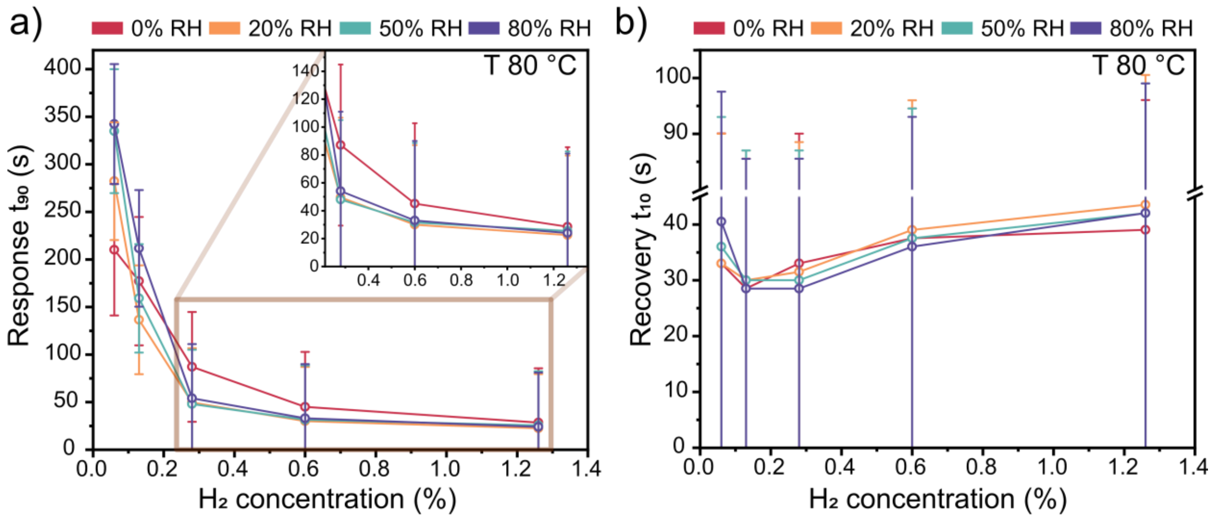

Figure S13: *a) t_{90} response times and b) t_{10} recovery times for the Pt sensor measured at the optimal operating temperature of 80 ºC and for 0%, 20%, 50%, and 80% RH. The sensor was measured at 0.06, 0.13, 0.28, 0.6, 1.26 vol.% H_2.*

Figure S13 shows the response/recovery times of the fabricated Pt sensor. The values are derived from a separate measurement, where the protocol followed is similar to the sensor performance measurements (***c.f.*** **Figure 3,** ***c.f.*** **Figure S5,** more details in **Methods -** *Sensor performance measurements*), with the difference that we have now allowed for more time during H_2

absorption/desorption. More specifically each H_2 pulse lasted for 900 seconds, followed by 1800 seconds of 100 vol.% synthetic air gas flow. This was done to ensure that the sensor reaches a stable state for all H_2 concentrations. It is important to emphasize that this measurement was conducted in the same flow reactor (more details in **Methods -** *Experimental Setup for sensor performance, humidity titration and long-term stability experiments*) with an effective volume of ~190 mL. Therefore, the response/recovery times are influenced by the gas-exchange time constant which is calculated to be (based on the gas flow used, i.e. 200 ml/min) 57 seconds. This means that intrinsic (=true) sensor responses faster or similar to this time constant are (heavily) convoluted by the gas exchange rate in the reactor, in particular due to hydrogen's high diffusivity, which leads to substantial concentration gradient inside the reactor (and the gas lines leading up to it). This effectively means that response/recovery times measured in this setup most likely overestimate (significantly) the true sensor response time as they don't represent the intrinsic response of the sensor. **Figure S13a)** depicts the response time (t_{90}) for the Pt sensor, at different RH levels (0, 20, 50, 80%) and H_2 concentrations (0.06 – 1.26 vol.%). At 0% RH the sensor exhibits a t_{90} of 210-28 seconds for the 0.06 – 1.26 vol.% range. When humidity is introduced, and at the first level (20% RH) the sensor is faster at almost all measured H_2 concentrations (apart from 0.06 vol.% H_2) with t_{90} = 282-22 seconds for the same range. As humidity is further increased to 50% RH, the sensor exhibits a response time of t_{90}=335-25 seconds, slower at all H_2 concentrations compared to 20% RH, but still faster than 0% RH above 0.13 vol.%. Finally, at the highest humidity (80% RH) the sensor exhibits a t_{90} = 342-24 seconds for the same H_2 concentration range, slower than 20% and 50% RH, however faster than 0% RH above 0.28 vol.% H_2. According to the ISO 26142:2010[33] specifications, the response/recovery time of a sensor should be: $t_{90} \leq 30$ sec (response time), and $t_{10} \leq 60$ sec (recovery time).Our sensor is able to meet the response time target at 1.26 vol.% H_2 for stationary applications for all measured RH levels. Additionally the sensor is able to meet the target at 0.6 vol.% for 20% RH (t_{90}= 30 sec) and barely misses the response time target at 0.6 vol.% for 50% and 80% RH (31 and 33 sec respectively). Due to the convoluted response of the sensor as explained previously, the reported response/recovery times are most likely an overestimation of the intrinsic response of the sensor. We are therefore confident that the sensor's response speed can be further improved, by either exploring the response times in a suitable experimental setup, or by employing alternative data analysis techniques, as has been previously established with a machine learning model tailored to accelerating a H2 sensor.[34, 35] **Figure S13b)** depicts the recovery time (t_{10}) for the Pt sensor, at different RH levels (0, 20, 50, 80%) and H_2 concentrations (0.06 – 1.26 vol.%). The sensor is able to meet the recovery time target ($t_{10} \leq 60$ sec) for all measured RH and H_2 concentrations.

Section 11: Selectivity/response in the presence of interfering gases

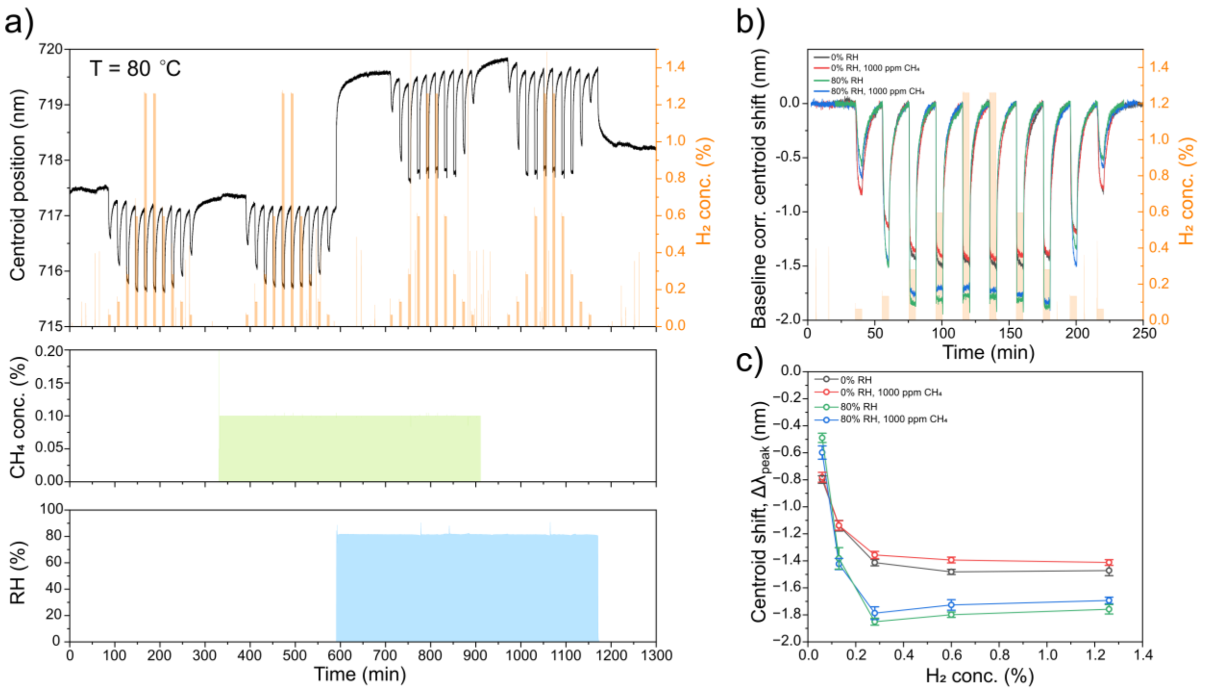

Figure S14: *Sensor response, with and without the presence of 1000 ppm of CH_4 and 80% RH, at T=80 ºC. a) Overall response (top), measured CH_4 concentration (middle) and measured RH (bottom) over time. b) Overlapped baseline-corrected centroid shift ($\Delta\lambda_{peak}$) for the 4 different H_2 pulse sets. c) Extracted centroid shift ($\Delta\lambda_{peak}$) over H_2 concentration for the 4 different H_2 pulse sets.*

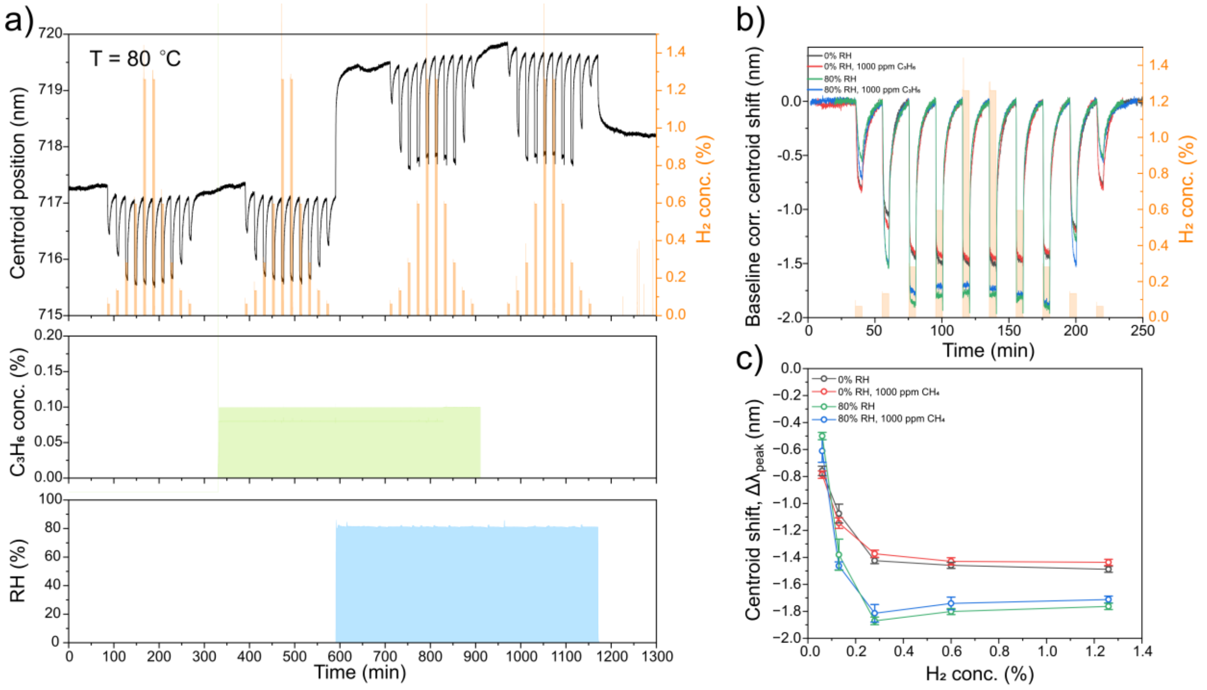

Figure S15: *Sensor response, with and without the presence of 1000 ppm of C_3H_6 and 80% RH, at T=80 ºC. a) Overall response (top), measured C_3H_6 concentration (middle) and measured RH (bottom) over time. b) Overlapped baseline-corrected centroid shift ($\Delta\lambda_{peak}$) for the 4 different H_2 pulse sets. c) Extracted centroid shift ($\Delta\lambda_{peak}$) over H_2 concentration for the 4 different H_2 pulse sets.*

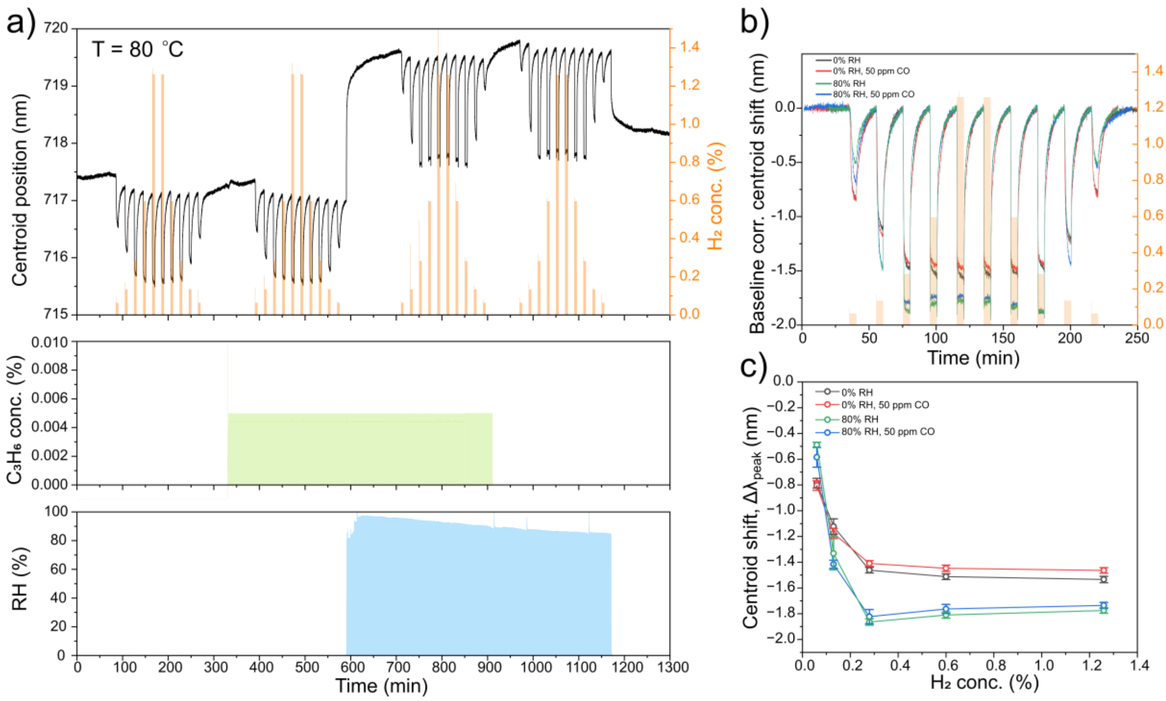

Figure S16: *Sensor response, with and without the presence of 50 ppm of CO and 80% RH, at T=80 ºC. a) Overall response (top), measured CO concentration (middle) and measured RH (bottom) over time. b) Overlapped baseline-corrected centroid shift ($\Delta\lambda_{peak}$) for the 4 different H_2 pulse sets. c) Extracted centroid shift ($\Delta\lambda_{peak}$) over H_2 concentration for the 4 different H_2 pulse sets.*

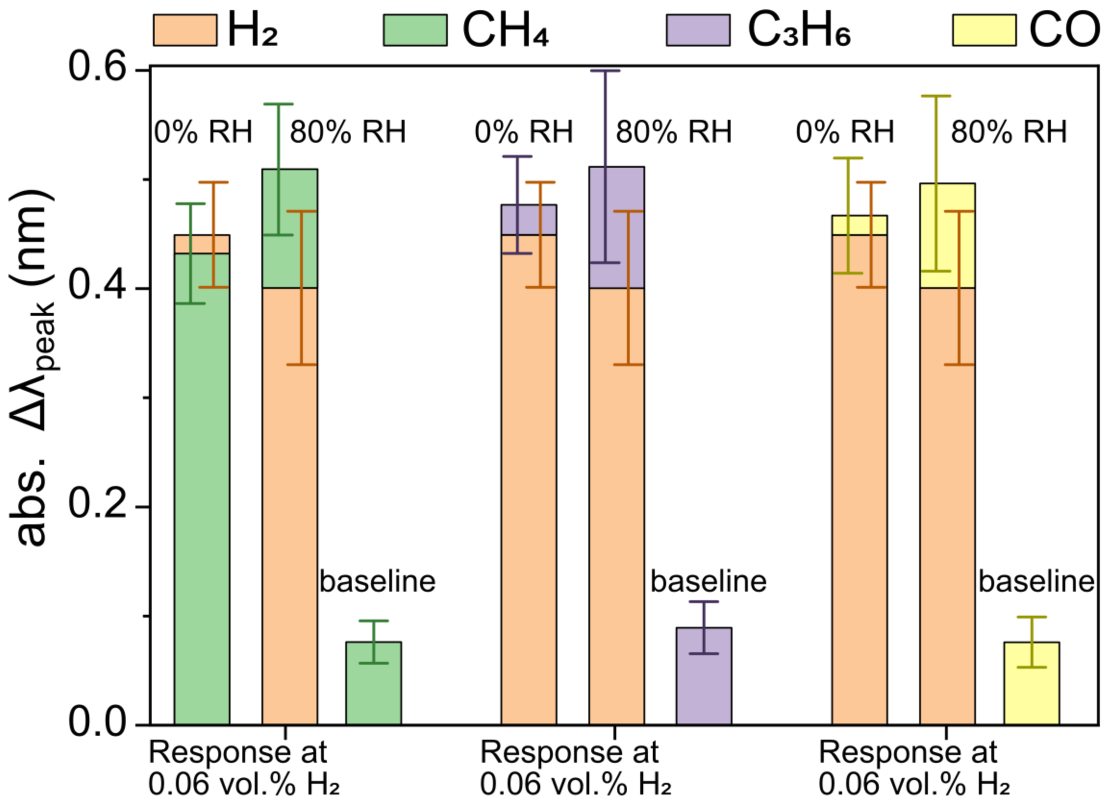

Figure S17: *Grouped bar graph comparing the absolute $\Delta\lambda_{peak}$ at the lowest measured H_2 concentration (0.06 vol.%) with and without interfering gases (CH_4, C_3H_6, CO), in 0% and 80% RH. The first bar in each group compares the response to 0.06 vol.% H_2 in 0% RH, the second bar in 80% RH, and the third bar denotes the baseline change from air to air+interfering gas, when the latter is first introduced. The H_2 bars show the response of the sensor without the presence of an interfering gas.*

The effect of interfering gases on the sensor's response has also been investigated and reported in **Figures S14, S15, S16**. Three different gases have been investigated, namely CH_4, CO, and C_3H_6. We have selected the first two species to follow the ISO 26142:2010[33] standard protocol for selectivity measurements. C_3H_6 was selected as another example of a volatile organic compound (VOC) that we have available in our lab and that was compatible with our experimental setup. It is worth mentioning that the ISO 26142:2010 standard protocol for selectivity requires measurements for concentrations of 5 ppm for CO and CH_4. Since we are unable to reach these low concentrations for the contaminants in our experimental setup, we opted for higher concentrations as follows: We chose a concentration of 1000 ppm for CH_4 and C_3H_6 as the suggested maximum concentration from The National Institute for Occupational Safety and Health (NIOSH),[36, 37] and 50 ppm for CO, suggested by the Occupational Safety and Health Administration (OSHA).[38] The measurement protocol starts with an initialization stage (see **Methods** - *Sensor performance measurements* in the main text for more details). During the

core stage, the sensor is exposed to 4 identical H_2 pulse sets (with the same H_2 concentrations as in the sensor performance measurements) in 4 different scenarios in the following order: i) 0% RH, no interfering gas, ii) 0% RH with interfering gas, iii) 80% RH with interfering gas, and iv) 80% RH, no interfering gas. **Figure S17** shows a comparison between the response of the sensor to 0.06 vol.% H_2 with and without an interfering gas, as well as the baseline shift of the sensor with a change of the background gas from air to air+interfering gas. For the latter, the baseline shift is small, compared to the shift of the peak during H_2 exposure, complying with the ISO 26142:2010 standard. In dry conditions (0% RH) the response to 0.06 vol.% H_2 in the presence of C_3H_6 and CO is slightly larger compared to the response in the absence of these interfering gases. More specifically an absolute shift of 0.48 nm for H_2+ C_3H_6 can be observed, as opposed to 0.45 nm for H_2 only. Similarly, an absolute shift of 0.47 nm is observed for H_2+CO. In the presence of CH_4 the response to 0.06 vol.% H_2 is slightly smaller compared to the response in the absence of it (0.43 nm vs 0.45 nm). Moving to the second lowest H_2 concentration (0.13 vol. %) (***c.f.*** **Figures S14c, S15c, S16c**) the response is again larger in the presence of all interfering gases, in dry conditions. As the H_2 concentration increases the response now becomes slightly smaller in the presence of the interfering gases. Interestingly, in highly humid conditions (80% RH) and for the lowest H_2 concentration there is a larger increase in the response in the presence of all interfering gases, as seen in **Figure S17**. The trend is similar to the dry conditions, with an enhanced response up to 0.13 vol.% H_2 and a small deterioration of the response for higher H_2 concentrations (***c.f.*** **Figures S14c, S15c, S16c**). These results showcase the substantial poisoning resistance of the sensor, especially in the low H_2 concentration regime, useful in early leak detection in dry but also highly humid environments.

Section 12: Long-term stability measurement – Baseline drift

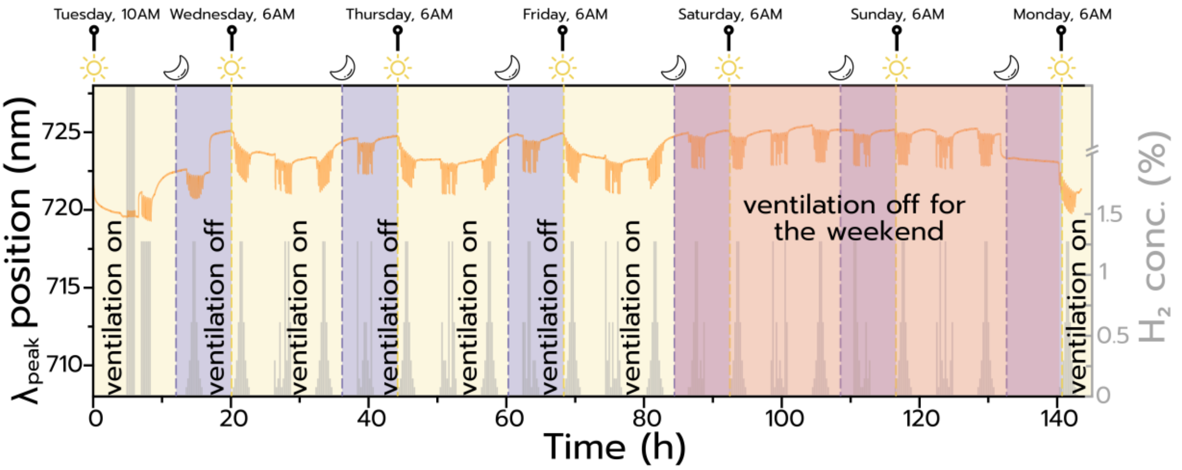

Figure S18: *Adjusted panel b) from* ***Figure 6*** *in the main text. The repeatable baseline drift is attributed to changes in the ventilation conditions during the measurement. During the night and weekend, the ventilation in the lab is turned off, which affects the baseline signal. Yellow rectangles denote daytime, where the ventilation is in full operation (6AM – 10PM). Purple rectangles denote nighttime, where the*

ventilation is turned off (10PM – 6AM). During the weekend, regardless of daytime/nighttime, the ventilation is off, consistent with the stable baseline that is visible in the measurement.

The first 86 hours, where the 3 baseline oscillations occur, take place during weekdays (**Figure S18**). During this time, there are 3, 18-hour long baseline drifts that occur during the day, as denoted by the yellow rectangles, where at the end of this period ventilation is turned off for the night. Looking at the end of the first baseline drift (36 hour-mark) we see that the measurements proceeds with a somewhat stable baseline for the next ~8 hours (until the ~44 hour-mark). That corresponds to the measurement continuing over the night, before room ventilation is turned on again the following morning (6AM), and thus repeating the cycle again. This broad oscillating drift ends at the ~86 hour-mark, which corresponds to a Friday evening. After that, and for the next 48 hours (weekend), ventilation is off leading to a stable baseline.

Section 13: Pt/Pd comparison

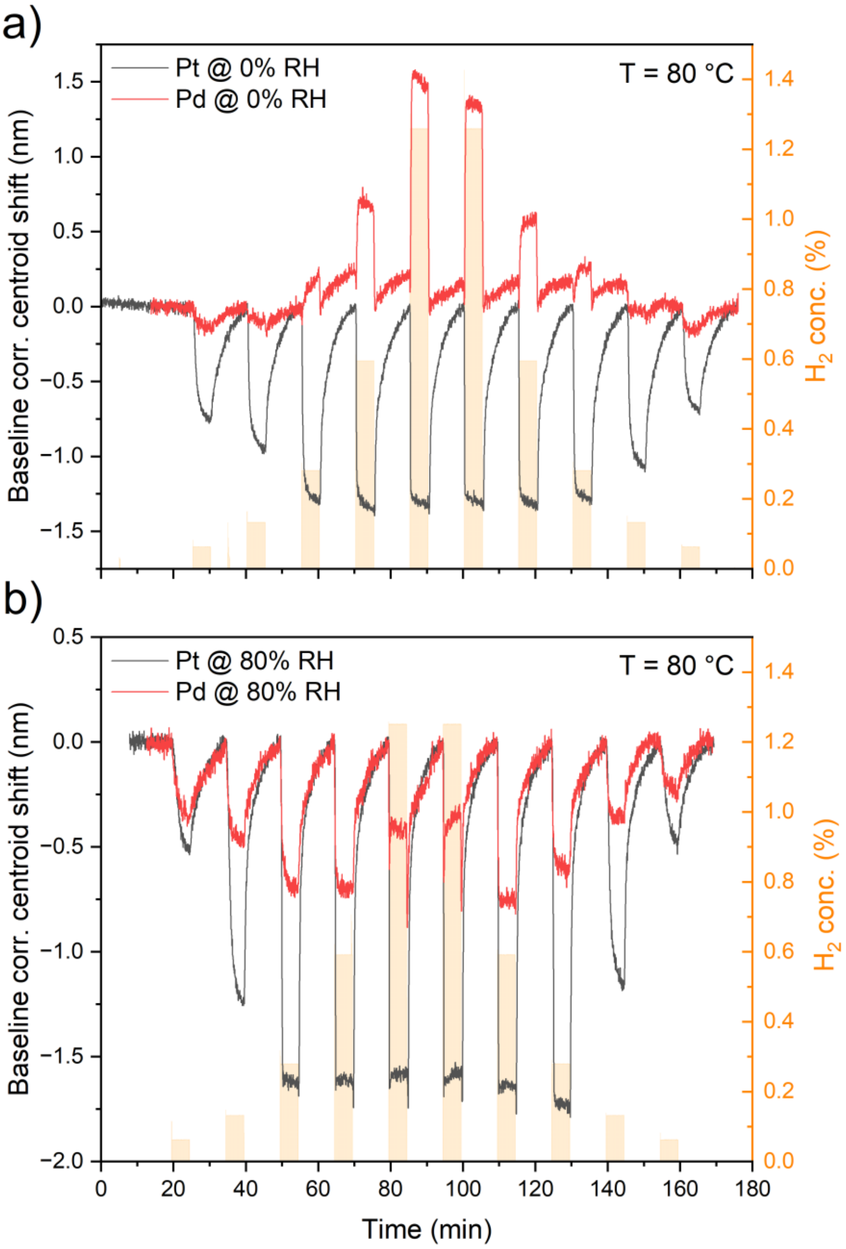

Figure S19: *Comparison between a Pt and a Pd nanoplasmonic sensor (same nominal particle dimensions). a) The response of Pt and Pd at 0% RH and b) at 80% RH. The samples were measured at the same time, under identical conditions, at 80 ºC. The H_2 concentration dependent blue-shift/red-shift of the Pd sensor stems from the competing surface (HOR) and bulk (H_2 absorption) reactions, resulting in a complex and relatively weak response, and thus rendering Pt a more suitable candidate for H_2 detection in high humidity environments.*

Section 14: Comparison of Pt/Pd based H_2 sensors in humid environments

Table S1: *Comparison of Pt/Pd based H_2 sensors in humid environments*

Materials	Sensing method	Operating Temperature (°C)	Background gas	Tested humidity range (%)	LoD investigation performed in humid environments*	LoD at highest RH**
*Pt NPs (**this work**)*	*Nanoplasmonic*	*80*	*Air*	*0-80*	*Yes*	*0.005 vol.%*
Pt NPs[17]	*Thermocatalytic*	*RT*	*Air*	*0-98*	*Yes*	*3 vol.%*
Pd/Mg thin film[39]	*Resistance*	*RT*	*Vacuum*	*0-80*	*No*	*0.5 bar*
Pd NCs[40]	*Resistance*	*RT*	*Air*	*0-81*	*No*	*1 vol.%*
Pd/V_2O_5 thin film[41]	*Resistance*	*100*	*Air*	*0-60*	*Yes*	*0.01 vol.%*
ITO-PdNi/Pt[42]	*Current*	*RT*	*Air*	*0-60*	*Yes*	*0.01 vol.%*
SnO_2/In_2O_3 thin films[43]	*Resistance*	*350*	*Air*	*20-95*	*Yes*	*0.0025 vol.%*
Pt NWs[24]	*Resistance*	*200*	*Air*	*0-50*	*No*	*0.1 vol.%*
PdAu NPs[7]	*Nanoplasmonic †*	*80*	*Air*	*0-80*	*Yes*	*0.01 vol.%*
LIG-Pt[25]	*Resistance*	*RT*	*Air*	*0-75*	*No*	*1 vol.%*
Pt-Pd NWs[26]	*Resistance*	*RT*	*Air*	*NA*	*No*	*NA*
Pt-PdO NWs[27]	*Resistance*	*RT*	*Air*	*NA*	*No*	*NA*
Pt NWs[23]	*Resistance*	*RT*	*Air/N_2*	*NA*	*No*	*NA*
Pt/Pd NW[22]	*Resistance*	*RT*	*Air*	*NA*	*No*	*NA*

**An LoD investigation in humid environments is defined as a measurement that includes varying H_2 concentrations in RH≠0%. ** The LoD at the highest RH (≠0%) investigated is defined as the lowest H_2 concentration (even if only 1 H_2 concentration was investigated) for which there is a sensor response. † Nanoplasmonic sensor with neural network-based data treatment.*

References

(1) Darmadi, I.; Nugroho, F. A. A.; Langhammer, C. High-Performance Nanostructured Palladium-Based Hydrogen Sensors - Current Limitations and Strategies for Their Mitigation. *ACS Sensors* **2020**, *5* (11), 3306-3327.

(2) Zoric, I.; Larsson, E. M.; Kasemo, B.; Langhammer, C. Localized surface plasmons shed light on nanoscale metal hydrides. *Adv Mater* **2010**, *22* (41), 4628-4633.

(3) Poyli, M. A.; Silkin, V. M.; Chernov, I. P.; Echenique, P. M.; Muiño, R. D.; Aizpurua, J. Multiscale Theoretical Modeling of Plasmonic Sensing of Hydrogen Uptake in Palladium Nanodisks. *The Journal of Physical Chemistry Letters* **2012**, *3* (18), 2556-2561.

(4) Tittl, A.; Kremers, C.; Dorfmüller, J.; Chigrin, D. N.; Giessen, H. Spectral shifts in optical nanoantenna-enhanced hydrogen sensors. *Opt. Mater. Express* **2012**, *2* (2), 111-118.

(5) Ekborg-Tanner, P.; Rahm, J. M.; Rosendal, V.; Bancerek, M.; Rossi, T. P.; Antosiewicz, T. J.; Erhart, P. Computational design of alloy nanostructures for optical sensing of hydrogen. *ACS Applied Nano Materials* **2022**, *5* (8), 10225-10236.

(6) Zhao, Z.; Knight, M.; Kumar, S.; Eisenbraun, E. T.; Carpenter, M. A. Humidity effects on Pd/Au-based all-optical hydrogen sensors. *Sensors and Actuators B: Chemical* **2008**, *129* (2), 726-733.

(7) Tomeček, D.; Moberg, H. K.; Nilsson, S.; Theodoridis, A.; Darmadi, I.; Midtvedt, D.; Volpe, G.; Andersson, O.; Langhammer, C. Neural network enabled nanoplasmonic hydrogen sensors with 100 ppm limit of detection in humid air. *Nature Communications* **2024**, 1-15.

(8) Anker, J. N.; Hall, W. P.; Lyandres, O.; Shah, N. C.; Zhao, J.; Van Duyne, R. P. Biosensing with plasmonic nanosensors. *Nature materials* **2008**, *7* (6), 442-453.

(9) Mayer, K. M.; Hafner, J. H. Localized surface plasmon resonance sensors. *Chem Rev* **2011**, *111* (6), 3828-3857.

(10) Taylor, A. B.; Zijlstra, P. Single-molecule plasmon sensing: current status and future prospects. *ACS sensors* **2017**, *2* (8), 1103-1122.

(11) Dahlin, A. B.; Tegenfeldt, J. O.; Höök, F. Improving the Instrumental Resolution of Sensors Based on Localized Surface Plasmon Resonance. *Analytical Chemistry* **2006**, *78* (13), 4416-4423.

(12) *Next-Gen hydrogen detector for low-oxygen environments*. https://www.insplorion.com/en/hydrogen-sensor-p2/ (accessed 29/09/2025).

(13) Klockar, P. E. R. AN OPTICAL MEASUREMENT DEVICE. EP EP 4241067 B1, 2025.

(14) Gland, J. L.; Sexton, B. A.; Fisher, G. B. Oxygen interactions with the Pt(111) surface. *Surface Science* **1980**, *95* (2-3), 587-602.

(15) Fassihi, M.; Zhdanov, V. P.; Rinnemo, M.; Keck, K. E.; Kasemo, B. A Theoretical and Experimental Study of Catalytic Ignition in the Hydrogen-Oxygen Reaction on Platinum. *Journal of Catalysis* **1993**, *141* (2), 438-452.

(16) Chase, M. W. NIST-JANAF Thermochemical Tables 4th ed. *J. of Physical and Chemical Reffernce Data* **1998**, 1529-1564.
(17) Geng, X.; Li, S.; Heo, J.; Peng, Y.; Hu, W.; Liu, Y.; Huang, J.; Ren, Y.; Li, D.; Zhang, L.; et al. Grain-Boundary-Rich Noble Metal Nanoparticle Assemblies: Synthesis, Characterization, and Reactivity. *Advanced Functional Materials* **2022**, *32* (34).
(18) Symons, E. A. Catalytic gas sensors. In *Gas Sensors: Principles, Operation and Developments*, Springer, 1992; pp 169-185.
(19) Lee, E.-B.; Hwang, I.-S.; Cha, J.-H.; Lee, H.-J.; Lee, W.-B.; Pak, J. J.; Lee, J.-H.; Ju, B.-K. Micromachined catalytic combustible hydrogen gas sensor. *Sensors and Actuators B: Chemical* **2011**, *153* (2), 392-397.
(20) Han, C.-H.; Hong, D.-W.; Kim, I.-J.; Gwak, J.; Han, S.-D.; Singh, K. C. Synthesis of Pd or Pt/titanate nanotube and its application to catalytic type hydrogen gas sensor. *Sensors and Actuators B: Chemical* **2007**, *128* (1), 320-325.
(21) Brauns, E.; Morsbach, E.; Kunz, S.; Bäumer, M.; Lang, W. A fast and sensitive catalytic gas sensors for hydrogen detection based on stabilized nanoparticles as catalytic layer. *Sensors and Actuators, B: Chemical* **2014**, *193*, 895-903.
(22) Yang, F.; Donavan, K. C.; Kung, S. C.; Penner, R. M. The surface scattering-based detection of hydrogen in air using a platinum nanowire. *Nano Letters* **2012**, *12* (6), 2924-2930.
(23) Yoo, H.-W.; Cho, S.-Y.; Jeon, H.-J.; Jung, H.-T. Well-Defined and High Resolution Pt Nanowire Arrays for a High Performance Hydrogen Sensor by a Surface Scattering Phenomenon. *Analytical Chemistry* **2015**, *87* (3), 1480-1484.
(24) Cao, F.; Zhao, P.; Wang, Z.; Zhang, X.; Zheng, H.; Wang, J.; Zhou, D.; Hu, Y.; Gu, H. An Ultrasensitive and Ultraselective Hydrogen Sensor Based on Defect-Dominated Electron Scattering in Pt Nanowire Arrays. *Advanced Materials Interfaces* **2018**, *6* (1).
(25) Lim, M.; Kim, J. Y.; Kang, H.; Yun, T. W.; Cho, H.-B.; Choa, Y.-H. Room temperature-based hydrogen gas sensing over Laser-Induced Graphene electrode supported Pt nanoparticles for low LOD. *Sensors and Actuators Reports* **2024**, *8*, 100247.
(26) Kumar, A.; Zhao, Y.; Mohsenifard, S.; Maheshkar, V.; Thundat, T.; Swihart, M. T. Platinum Decorated Palladium Nanowires for Room-Temperature Hydrogen Detection. *Advanced Sensor Research* **2024**, *3* (8).
(27) Cho, H. J.; Chen, V. T.; Qiao, S.; Koo, W. T.; Penner, R. M.; Kim, I. D. Pt-Functionalized PdO Nanowires for Room Temperature Hydrogen Gas Sensors. *ACS Sensors* **2018**, *3* (10), 2152-2158.
(28) Tiburski, C.; Boje, A.; Nilsson, S.; Say, Z.; Fritzsche, J.; Strom, H.; Hellman, A.; Langhammer, C. Light-off in plasmon-mediated photocatalysis. *ACS nano* **2021**, *15* (7), 11535-11542.
(29) Bu, Y.; Niemantsverdriet, J. H.; Fredriksson, H. O. Cu model catalyst dynamics and CO oxidation kinetics studied by simultaneous in situ UV–Vis and mass spectroscopy. *ACS Catalysis* **2016**, *6* (5), 2867-2876.

(30) Baffou, G.; Berto, P.; Bermúdez Ureña, E.; Quidant, R.; Monneret, S.; Polleux, J.; Rigneault, H. Photoinduced Heating of Nanoparticle Arrays. *ACS Nano* **2013**, *7* (8), 6478-6488.
(31) Antosiewicz, T. J.; Apell, S. P.; Zach, M.; Zoric, I.; Langhammer, C. Oscillatory optical response of an amorphous two-dimensional array of gold nanoparticles. *Phys Rev Lett* **2012**, *109* (24), 247401.
(32) Larsson, E. M.; Alegret, J.; Käll, M.; Sutherland, D. S. Sensing Characteristics of NIR Localized Surface Plasmon Resonances in Gold Nanorings for Application as Ultrasensitive Biosensors. *Nano Letters* **2007**, *7* (5), 1256-1263.
(33) Standardization, I. O. f. Hydrogen detection apparatus — Stationary applications (ISO Standard No. 26142:2010). **2010**.
(34) Martvall, V.; Klein Moberg, H.; Theodoridis, A.; Tomeček, D.; Ekborg-Tanner, P.; Nilsson, S.; Volpe, G.; Erhart, P.; Langhammer, C. Accelerating Plasmonic Hydrogen Sensors for Inert Gas Environments by Transformer-Based Deep Learning. *ACS Sensors* **2025**.
(35) Lin, X.; Cheng, M.; Chen, X.; Zhang, J.; Zhao, Y.; Ai, B. Unlocking Predictive Capability and Enhancing Sensing Performances of Plasmonic Hydrogen Sensors via Phase Space Reconstruction and Convolutional Neural Networks. *ACS sensors* **2024**, *9* (8), 3877-3888.
(36) *Methane*. Pennsylvania Department of Health, https://www.pa.gov/content/dam/copapwp-pagov/en/health/documents/topics/documents/environmental-health/Methane.pdf#:~:text=The%20National%20Institute%20for%20Occupational%20Safety%20and,28%20mg/L%20pose%20a%20potential%20explosion%20risk. (accessed 14/10/2025).
(37) *Propane*. The National Institute for Occupational Safety and Health (NIOSH), https://www.cdc.gov/niosh/idlh/74986.html (accessed 14/10/2025).
(38) *Carbon monoxide*. Occupational Safety and Health Administration (OSHA), https://www.osha.gov/laws-regs/regulations/standardnumber/1917/1917.24 (accessed 14/10/2025).
(39) Sanger, A.; Kumar, A.; Chauhan, S.; Gautam, Y. K.; Chandra, R. Fast and reversible hydrogen sensing properties of Pd/Mg thin film modified by hydrophobic porous silicon substrate. *Sensors and Actuators B: Chemical* **2015**, *213*, 252-260.
(40) Yaqoob, U.; Uddin, A. S. M. I.; Chung, G. S. Foldable hydrogen sensor using Pd nanocubes dispersed into multiwall carbon nanotubes-reduced graphene oxide network assembled on nylon filter membrane. *Sensors and Actuators B: Chemical* **2016**, *229*, 355-361.
(41) Sanger, A.; Kumar, A.; Kumar, A.; Jaiswal, J.; Chandra, R. A fast response/recovery of hydrophobic Pd/V2O5 thin films for hydrogen gas sensing. *Sensors and Actuators B: Chemical* **2016**, *236*, 16-26.
(42) Rahamim, G.; Mirilashvili, M.; Nanikashvili, P.; Greenberg, E.; Shpaisman, H.; Grinstein, D.; Welner, S.; Zitoun, D. Hydrogen sensors with high humidity tolerance based on indium-tin oxide colloids. *Sensors and Actuators, B: Chemical* **2020**, *310* (December 2019), 127845-127845.
(43) Murthy, A. S. R.; Gnanasekar, K. I.; Jayaraman, V. Hydrogen sensing behavior of SnO2and in2O3thin films in wide range of humid conditions-An exploratory data analysis. *Journal of Environmental Chemical Engineering* **2020**, *8* (5), 104158-104158.
